# AURKB-driven dissolution of CIZ1–RNA assemblies from the inactive X chromosome in mitosis

**DOI:** 10.1093/nar/gkag018

**Published:** 2026-02-02

**Authors:** Lewis Byrom, Gabrielle L Turvey, Adam A Dowle, Megan Thomas, Navin Shirodkar, Ben J Green, Maxwell Brown, Charlotte Ball, Kate E Chapman, Elena Guglielmi, William Dickson, Emma Noon, Sajad Sofi, Justin F-X Ainscough, Alfred A Antson, Dawn Coverley

**Affiliations:** Mammalian cell cycle research group, Department of Biology, University of York, York, YO10 5DD, United Kingdom; York Biomedical Research Institute, University of York, York, YO10 5DD, United Kingdom; Mammalian cell cycle research group, Department of Biology, University of York, York, YO10 5DD, United Kingdom; York Biomedical Research Institute, University of York, York, YO10 5DD, United Kingdom; Metabolomics and Proteomics Laboratory, York Bioscience Technology Facility, University of York, York, YO10 5DD, United Kingdom; Mammalian cell cycle research group, Department of Biology, University of York, York, YO10 5DD, United Kingdom; Mammalian cell cycle research group, Department of Biology, University of York, York, YO10 5DD, United Kingdom; Mammalian cell cycle research group, Department of Biology, University of York, York, YO10 5DD, United Kingdom; Mammalian cell cycle research group, Department of Biology, University of York, York, YO10 5DD, United Kingdom; Mammalian cell cycle research group, Department of Biology, University of York, York, YO10 5DD, United Kingdom; Mammalian cell cycle research group, Department of Biology, University of York, York, YO10 5DD, United Kingdom; Mammalian cell cycle research group, Department of Biology, University of York, York, YO10 5DD, United Kingdom; York Biomedical Research Institute, University of York, York, YO10 5DD, United Kingdom; Mammalian cell cycle research group, Department of Biology, University of York, York, YO10 5DD, United Kingdom; Mammalian cell cycle research group, Department of Biology, University of York, York, YO10 5DD, United Kingdom; Mammalian cell cycle research group, Department of Biology, University of York, York, YO10 5DD, United Kingdom; Mammalian cell cycle research group, Department of Biology, University of York, York, YO10 5DD, United Kingdom; York Biomedical Research Institute, University of York, York, YO10 5DD, United Kingdom; York Structural Biology Laboratory, Department of Chemistry, University of York, York YO10 5DD, United Kingdom; Mammalian cell cycle research group, Department of Biology, University of York, York, YO10 5DD, United Kingdom; York Biomedical Research Institute, University of York, York, YO10 5DD, United Kingdom

## Abstract

Cip1-interacting zinc-finger protein 1 (CIZ1) interacts with *Xist* lncRNA to form large RNA–protein assemblies at the inactive X-chromosome (Xi) in female mammalian nuclei, plus smaller assemblies in both sexes. CIZ1 assemblies influence underlying chromatin, and their disruption alters the expression of autosomal and X-linked gene clusters. Here, we explore the regulated dissolution of CIZ1–Xi assemblies during mitosis and show that, like *Xist*, CIZ1 is released in prometaphase under the regulation of Aurora Kinase B (AURKB). The part of human/mouse CIZ1 comprising 179/181 C-terminal amino acids encodes a matrin-3 domain that facilitates dimerization to form a compact folded core with disordered C-terminal extensions. Mass spectrometry revealed 56 high-confidence interacting partners of the C-terminal fragment, predominantly chromatin, nuclear matrix, and RNA-binding proteins. Phosphomimetic mutation of three conserved AURKB sites in the C-terminal extensions released CIZ1 from its nuclear anchor points, but did not affect its interaction with chromatin or nuclear matrix proteins. In contrast, the same mutations, or deletion of the C-terminal extensions, abolished interaction with RNAs, including *Xist*. Together, the data suggest CIZ1 is a regulatable component of the protein–RNA assemblies that preserve epigenetic stability across the nucleus, and that AURKB drives their dissolution in mitosis via dissociation of CIZ1 from RNA.

## Graphical Abstract

**Graphical Abstract:**
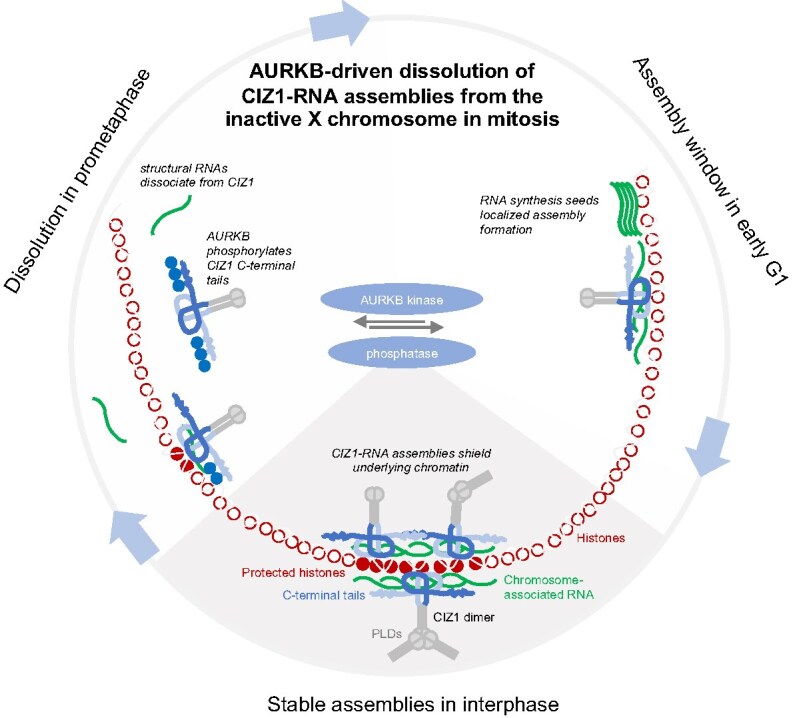

## Introduction

The mammalian cell nucleus is organized to facilitate tight control over gene expression—some genes are accessible, some inaccessible, and some poised to respond to changing cues. Control is achieved through associated proteins and their post-translational modifications, but also via non-coding RNAs that seed the formation of protein assemblies that are an integral part of the nuclear architecture, and crucial determinants of structure-based control. Historically, the proteins whose location within the nucleus remains unaffected by extraction of DNA and lipids were referred to as nuclear matrix proteins, whereas those that are sensitive to digestion of RNA are the RNA-dependent nuclear matrix [1, 2]. Many of these are part of RNA-seeded protein condensates and include those that assemble around the archetypal long non-coding RNA (lncRNA) *Xist* at the inactive X chromosome (Xi) in female mammalian cells [3]. RNA–protein particles (RNPs) have been implicated in all aspects of nuclear function, including RNA modification, processing, turnover, and localization, as well as in the control of gene expression. While most RNPs do not diffuse freely, there is no consensus on the extent to which they remain spatially isolated versus connected into a wider network. Fundamental questions remain around how a cell, in which chromatin is spatially and structurally organized according to its lineage, can collapse its nucleus and condense chromosomes at mitosis, and then reassemble accurate structural organization in daughter cells.

Our focus is Cip1-interacting zinc finger protein 1 (CIZ1), which meets the definition of a nuclear matrix protein in that it remains *in situ* in subnuclear foci even when chromatin is digested away [4]. CIZ1 subnuclear foci are evident in both sexes, but in females they also aggregate into supramolecular assemblies around the Xi, seeded by *Xist* lncRNA. In differentiated cells, in which *Xist*-dependent repression of Xi chromatin has been achieved, CIZ1 and *Xist* coexist within RNP particles, and their recruitment to Xi is co-dependent [5, 6]. Although CIZ1 is one of the most stable components of the multiprotein supramolecular assemblies (SMACs) that drive heterochromatinization of the Xi [7], neither the loss of *Xist* nor deletion of CIZ1 causes widespread derepression of X-linked genes once its repressed state has been established, yet both are required for accurate control over a subset of ‘escape’ genes. In the case of CIZ1, these are not limited to X-linked genes but encompass ~0.4% of the transcriptome in primary embryonic fibroblasts (PEFs) [8] distributed across all chromosomes, suggesting that the assemblies that are associated with autosomes perform a similar function to those at the Xi, likely as part of assemblies seeded by other lncRNAs.

**Figure 1.**
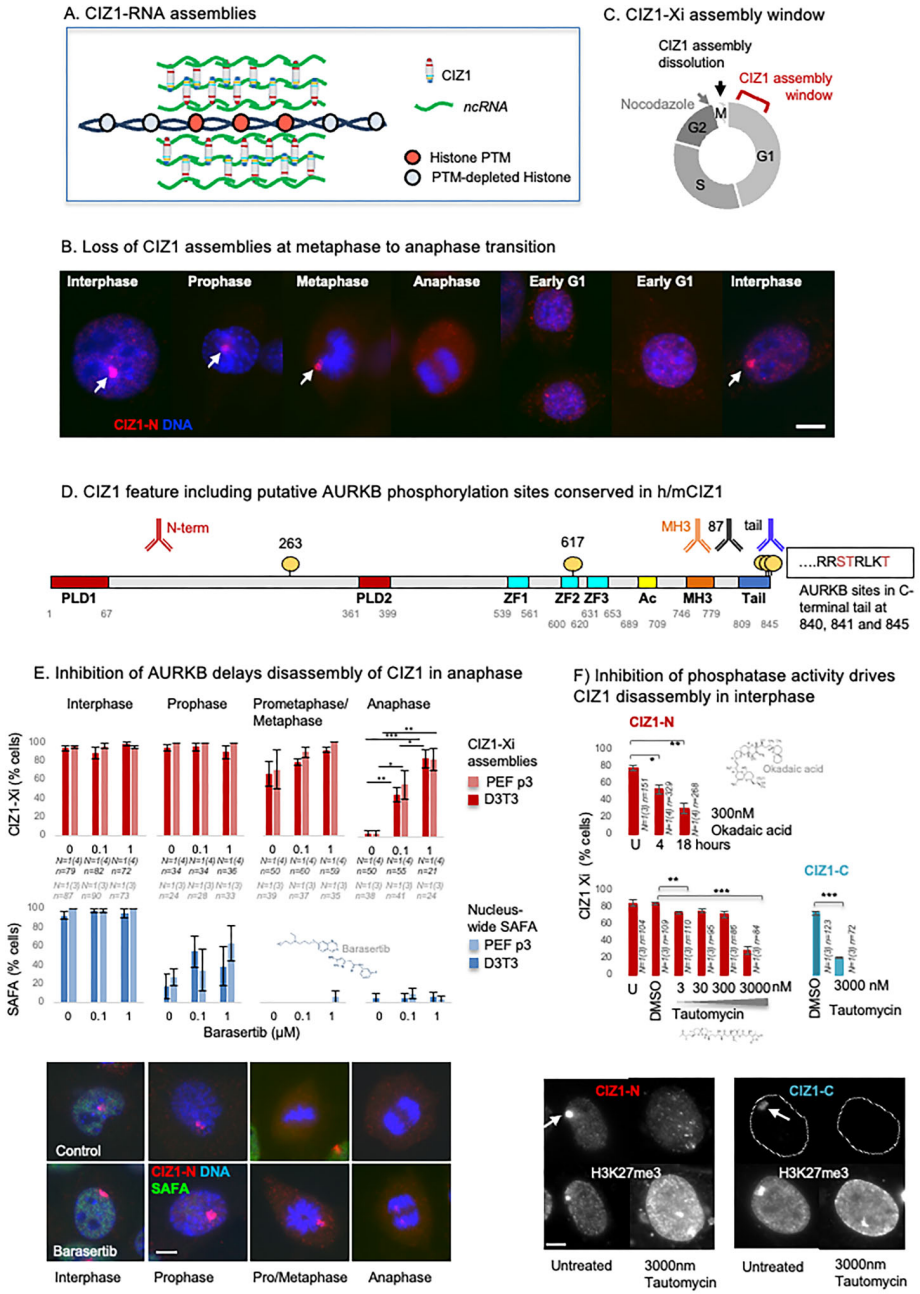
Dissolution of CIZ1–Xi assemblies in mitosis. (**A**) Model of CIZ1–RNA assemblies surrounding and protecting the modification status of underlying chromatin [9, 10]. (**B**) Illustrative immunofluorescence images of female murine D3T3 cells stained for CIZ1 via N-terminal epitopes (CIZ1-N, red, detected with pAb 1794), revealing large protein assemblies at the inactive X chromosome (white arrows) that are not detected in mitosis. DNA is shown in blue, bar is 5 µm. (**C**) Diagram illustrating loss in mitosis and the early G1 phase window during which reformation of CIZ1–Xi assemblies takes place, determined previously using cells that were synchronized in G1 phase by release from arrest with nocodazole [10]. (**D**) Map showing conserved putative AURKB phosphorylation sites between murine and human CIZ1 (circles) displayed on full-length murine CIZ1 (NP_082688.1.) The location of epitopes of CIZ1 antibodies used throughout is shown above. Conserved prion-like domains (PLD1 and PLD2) are in red [9], zinc fingers 1–3 in cyan (ZnF_C2H2 SM00355, ZF_C2H2 sd00020, and ZF_C2H2 sd00020), acidic region (Ac) in yellow, matrin-3 homology domain (MH3) in orange (ZnF_U1, smart00451), and h37/m38 amino-acid C-terminal tail in blue. The sequence context and identity of three conserved AURKB phosphorylation sites in the extreme C-terminus are shown. (**E**) Frequency of cells with CIZ1–Xi assemblies (red) or nucleus-wide SAFA (blue) in cells passing through the stages of mitosis indicated, for D3T3 cells and female primary embryonic fibroblasts (PEFs at p3), in the presence and absence of the AURKB kinase inhibitor barasertib [11] at 0.1 and 1 µM. Results show the average of 3–4 independent replicates within one experiment for each line, with SEM. *n* indicates the number of nuclei inspected (PEF grey, 3T3 black). Statistical analysis of CIZ1–Xi frequency in anaphase cells shows one-way ANOVA with Tukey post hoc test within each cell type, where * <.05, ** <.01, *** <.001. Below, example immunofluorescence images of D3T3 cells through mitosis, with and without 1 µM barasertib. Cells were stained for the N-terminal domains of CIZ1 (CIZ1-N, red) and SAFA (green). DNA is shown in blue, bar is 5 microns. (**F**) Upper histogram shows the proportion of cells with CIZ1-marked Xi in cycling populations of female D3T3 cells after the indicated times exposed to 300 nM Okadaic acid, visualized via CIZ1-N (red). Lower, histograms show the effect of the indicated concentrations of tautomycin for 15 h, stained for CIZ1-N or the ‘tail’ epitope in the C-terminal end of CIZ1 (CIZ1-C, rabbit pAb). Comparison of technical replicates is by t-test, where * <.05, ** <.01, *** <.001. Error bars show SEM. Below, example images showing H3K27me3-marked Xi chromatin in cells stained for CIZ1-N or CIZ1-C at 15 h with or without tautomycin. Bar is 5 microns.

CIZ1 interacts directly with *Xist*, preferring its E-repeat sequences [5, 6], but also interacts with other RNAs via at least two RNA interaction interfaces [9]. In the N-terminal half of CIZ1, RNA interaction is mediated by low-complexity prion-like domains (PLDs) that also drive self-association, and in the C-terminal third by as yet undefined sequences. Both are required for *de novo* formation of CIZ1 assemblies at Xi in fibroblasts and in the co-recruitment of *Xist* [9] (Fig. 1A). The requirement for multivalent interaction with RNA makes CIZ1–RNA assemblies susceptible to the effect of monovalent competitors, and assemblies can in fact be dissolved experimentally using dominant-negative fragments of CIZ1 (DNFs), with immediate (within days) effects on underlying chromatin and gene expression [10]. Unscheduled dissolution of CIZ1–RNA assemblies is now linked with early-stage breast cancers [10], so the molecular determinants of their normal assembly and disassembly within the nucleus are important to understand. Here, we explore the interactions that support anchorage and the regulated dissolution of CIZ1–Xi assemblies during mitosis in normal cells.

## Materials and methods

### Recombinant protein expression and purification

N-terminal GST-tagged CIZ1 constructs were expressed in BL21 RP CodonPlus cells (Agilent), grown in Luria broth supplemented with 100 µg/ml ampicillin at 37°C. Bacterial pellets were washed in PBS supplemented with 1 mM DTT, 3 mM ethylenediaminetetraacetic acid (EDTA), 1 mM PMSF (wash buffer), centrifuged at 4500 RPM, and the pellet snap frozen in liquid nitrogen. Pellets were thawed on ice and resuspended in wash buffer supplemented with EZblock protease inhibitor at a ratio of 5 ml buffer per 1 g *Escherichia coli*, then sonicated using a 6 mm diameter MS 73 microtip (Bandelin) and a SONOPULS ultrasonic homogenizer (Bandelin) operating at 60%–70% power. The supernatant was removed and then syringe filtered using a 0.45 µm filter before purification via GST affinity chromatography using ÄKTA pure™ (GE Healthcare) chromatography systems. Five millilitres of GSTrap 4B column (Cytiva) were equilibrated with 10 column volumes (cv) wash buffer before applying the supernatant at 0.5 ml/min. The column was washed with 10 cv wash buffer followed by 10 cv of cleavage buffer (50 mM Tris–HCl pH 7, 150 mM NaCl, 1 mM EDTA, 1 mM DTT), then injected with 1 cv of cleavage buffer supplemented with 0.4% v/v PreScission Protease (Cytiva) for on-column GST-tag cleavage at 4°C for 16 h. Untagged CIZ1 protein was eluted on ÄKTA with 2 cv, and the eluted protein was concentrated to ∼0.5 ml using Vivaspin^®^ 6, 10 kDa MWCO columns and then injected via a 0.5 or 1 ml loop onto a pre-equilibrated Superdex^®^ 200 Increase 10/300 GL column (Cytiva). Protein was separated over 1 cv at 0.5 ml/min, collecting 0.5 or 1 ml elution fractions. Peak fractions were identified using absorbance at 280 nm and were pooled and concentrated before snap freezing in liquid nitrogen for storage at −80°C. NanoDrop spectrophotometer readings were used to measure final protein concentration and purity was verified by sodium dodecyl sulfate–polyacrylamide gel electrophoresis (SDS–PAGE).

To prepare bait protein for interaction studies, expression of GST-tagged C-terminal CIZ1 fragments or GST controls was carried out as above, with resuspension of cells in cleavage buffer supplemented with EZblock protease inhibitor and 1 mM PMSF. Clarified supernatants (∼40 ml) were incubated with Glutathione Sepharose 4B bead slurry (Cytiva) at 4°C for 1 h in the presence of 0.8 µl Benzonase (Millipore) and 8 µl RNAse (Roche). GST-CIZ1-coated beads were extensively washed in cleavage buffer followed by isotonic buffer before addition of nuclear extracts.

### SEC-MALLS

One hundred microlitres of >1 mg/ml purified protein was analysed using a Superdex^®^ S200 10/300 GL column (GE Healthcare), equilibrated in cleavage buffer with a flow rate of 0.5 ml/min using a Shimadzu HPLC system (SPD-20A UV detector, LC20-AD isocratic pump system, DGU-20A3 degasser, and SIL-20A autosampler) at room temperature (20 ± 2°C). Detection of light scattering was carried out using a Wyatt HELEOS-II multi-angle light scattering detector and a Wyatt rEX refractive index detector. Shimadzu LabSolutions software was used to control the HPLC and Astra 7 software for the HELEOS-II and rEX detectors. MWs were estimated using the Zimm fit method with degree 1, using the Astra 7 software. A value of 0.182 was used for protein refractive index increment (dn/dc).

### Cells, culture, and transfection

Primary embryonic fibroblasts (PEFs), D3T3 murine fibroblast cell line [12], and HeLa cells [13] (Table 1) were cultured as adherent populations in High Glucose DMEM GlutaMAX (Gibco), supplemented with 10% foetal bovine serum (PAA) and 1% pen/strep/glutamine (Gibco) at 37°C, 5% CO_2_. PEFs were generated from C57BL/6 mice embryos at day 13–14 of gestation. CIZ1 null mice were generated from ES clone (IST13830B6, TIGM) using a neomycin resistance gene trap downstream of exon 1, as described previously [5]. Heterozygous CIZ1^±^ mice were bred to generate CIZ1^+/+^ (wild-type) mice and CIZ1^−/−^ (CIZ1 null mice), and CIZ1 status was confirmed for parents and embryo-derived primary cells by a combination of polymerase chain reaction (PCR) genotyping, immunoblot, and immunofluorescence to detect CIZ1 protein. Cells utilized for transfection and immunofluorescence assays were plated onto glass coverslips and used at ~80% confluence. Transfection reactions were carried out using TransIT-X2 (Mirus) for 24 or 48 h as indicated.

**Table 1.** Cells

Cell type	Strain ID	CIZ1 Genotype	Sex
	Cell Lines		
Murine fibroblast	D3T3		Female
Human carcinoma	HeLa		Female
	Primary cell isolates		
PEF	13.49	WT	Female
PEF	13.47	WT	Male
PEF	35.2A	KO	Female
PEF	13.66	KO	Female
PEF	13.54	KO	Male
PEF	13.85	KO	Male
PEF	13.79	WT	Female
PEF	13.67	KO	Female
PEF	13.55	KO	Female
PEF	13.37	WT	Female
PEF	45.1fb	WT	Male
PEF	16.5de	KO	Male
PEF	13.74	KO	Female
PEF	13.54	KO	Male
PEF	13.84	WT	Male

### Retrieval of binding proteins from nuclear extracts

HeLa cells at 90% confluency were washed in PBS and then rinsed in cold isotonic buffer (50 mM Hepes pH 7.8, 5 mM MgCl_2_, 5 mM potassium acetate, 135 mM NaCl) with protease inhibitor (Roche). Cells in minimal buffer were scrape harvested at 4°C and disrupted by Dounce homogenization until nuclei were released. Protein fractions were extracted via centrifugation to separate insoluble (nuclei and cytoskeleton) and soluble fractions. The insoluble fraction was resuspended in an equivalent volume of isotonic buffer supplemented with 400 mM NaCl for 10 min on ice, then centrifuged to generate a second supernatant fraction. The pellet was resuspended in a half-equivalent volume of 800 mM NaCl and then centrifuged to generate a third supernatant fraction. Soluble protein fractions collected in 400 and 800 mM NaCl were pooled, and diluted to a final concentration of 135 mM NaCl before incubation with GST-CIZ1 beads for 2 h at 4°C rotating at 7 rpm. Beads were washed five times in isotonic buffer before snap freezing. Study A compared GST-C179_WT_ to GST control. Study B compared GST-C179_WT_ to GST-C179Δ38 and GST control. Study C compared GST-C179_WT_ to GST-C179_DDD_ and GST control.

### Immunofluorescence assays

Cells grown on coverslips were washed in D-PBS, followed by a brief wash with cytoskeletal buffer (CSK, 10 mM PIPES pH 6.8, 100 mM NaCl, 300 mM sucrose, 1 mM sucrose, 1 mM MgCl_2_, 1 mM EGTA), supplemented with 0.1% Triton X-100 where indicated. Cells were fixed with 4% w/v paraformaldehyde (PFA) for 15 min, washed, then incubated in BSA antibody buffer (PBS supplemented with 0.1% BSA, 0.02% SDS, and 0.1% Triton X-100). Primary antibodies (Table 3) were diluted in BSA antibody buffer and incubated with cells on coverslips for 1 h at 37°C, washed, and then incubated with secondary antibodies. Coverslips were mounted onto glass slides with DAPI-containing Vectashield (Vector Laboratories). For analysis of mitotic cells coverslips were fixed in PFA without prior wash steps.

### Microscopy

All images were taken using a 63×/1.40 plan-apochromat objective and Zeiss filter sets 2, 10, and 15 (G365 FT395 LP420, BP450-490 FT510 BP-515-565, and BP546/12 FT580 LP590). Image acquisition was carried out using an Axiocam 506 mono camera (Zeiss) and Axiovision software (SE64 version 4.9.1). Within experiments where fluorescence intensities were quantified, exposures were consistent across a set of conditions, and measurements were made from unedited raw images. Images were analysed and prepared for figures using FIJI.

Live cell imaging to quantify morphological differences between WT and CIZ1-null PEFs (passage 1) over the cell cycle was conducted using LiveCyte 2 (PhaseFocus). Cells were seeded into 24-well glass bottom plates (Cellvis) a day prior to imaging of two wells per cell population across three fields of view (1000 µm), using a 10× Plan N objective. Exposure time was set at 25 ms at 100% power, with slice count of 1 and slice spacing of 5.0 µm. Images were taken at 6 min intervals over 24 h. Calculation of the number of cells entering mitosis and then successfully dividing over a time window was made by identifying cells that brighten and round up and inspecting before and after this point in time. Entry into mitosis was determined by counting the total number of cells in the first frame and then calculating the total number of mitotic cells observed as a percentage. Successful division was determined visually, generating three categories: normal (a clean division into two equally sized daughters), abnormal (an aberrant division into two irregular or asymmetric daughters), and failed (flattening of mitotic cells without division).

### RNA probe generation

*Xist* digoxygenin (DIG)-labelled RNA probes were synthesized by PCR from pCMV-Xist-PA (Addgene) [14], using primers in Table 2 to incorporate a T7 promoter sequence. GADPH RNA probe was generated from pTRI-GAPDH-Mouse control plasmid (Invitrogen Northern Max kit), described previously [9]. PCR products (3–4 μg) were used directly for *in vitro* transcription with MEGAshortscriptTM Transcription Kit (Ambion). TURBO DNase was added to completed reactions to remove DNA template. DIG-labelled RNA probes were purified from transcription reactions using MEGAclear Transcription Clean-Up Kit (Ambion). RNA was collected in 2 × 40 μl elutions, using preheated (95°C) buffer and quantified by absorbance at 260 nm.

**Table 2.** Primers

Variant created	Primer for mutagenesis
GST-C179_Δtail_	FP CTTTGACAGCCCTGTTCTGATAACTCGAGCGGC
	RP GCCGCTCGAGTTATCAGAACAGGGCTGTCAAAG
	FP CCACTACCTCGGCGCGATGACCGCCTCAAAGACTGATAACTCGAGCGG
GST-C179_DDD_	RP CCGCTCGAGTTATCAGTCTTTGAGGCGGTCATCGCGCCGAGGTAGTGG
GFP-m845_DDD_GFP-C275_DDD_	FP CTCCCCTTCGGCGCGATGACCGCCTCAAAGACTGATAGAGGGAGCTC
	RP GAGCTCCCTCTATCAGTCTTTGAGGCGGTCATCGCGCCGAAGGGGAG
GFP-m845_AAA_	FP CTCCCCTTCGGCGCGCTGCTCGCCTCAAAGCTTGATAGAGGGAGCTC
	RP GAGCTCCCTCTATCAAGCTTTGAGGCGAGCAGCGCGCCGAAGGGGAG
GST-C181_ΔMH3_	FP GGATTTCCTGGTGCCAGTGATGAAAGCCAAGAACCCAAGC
	RP GCTTGGGTTCTTGGCTTTCATCACTGGCACCAGGAAATCC
GST-C181_Δ37_	FP CCTGACTGCACTGTTCTGATAGAGGGAGC
	RP GCTCCCTCTATCAGAACAGTGCAGTCAGG
GST-C181_ΔAcD_	FP GCTTTGAGAGTGGTCAATTCTGCAAGCAGGTGAAGC
	RP GCTTCACCTGCTTGCAGAATTGACCACTCTCAAAGC
	Primers for amplification
pCMV-Xist-PA	FP TAATACGACTCACTATAGGGAGCACTAGCTATGGCTCTCTG
	RP CACATAACACACATGCACACACGC
Vector	Primers for sequencing
pGEX-5	CTG GCA AGC CAC GTT TGG
pGEX-3	GGA GCT GCA TGT GTC AGA GG
pEGFP for	TTT AGT GAA CCG TCA GAT C
pEGFP rev	TTT AAA GCA AGT AAAACC TC

### Electrophoretic mobility shift assays

Purified proteins in binding buffer [10 mM Tris–HCl pH 7 at 25°C, 30 mM NaCl, 2.5 mM MgCl_2_, 0.1% IGEPAL CA-630, 0.1 mg/ml yeast tRNA, 1.5% RNase OUT (Invitrogen), 0.2 mM EDTA, and 0.2 mM DTT] were heated to 30°C for 20 min. RNA probes were diluted and denatured at 80°C for 3 min before snap cooling on ice and used at 0.66 nM for Xist repeat E, 0.65 nM for GAPDH in incubations with proteins for 20 min at 30°C. Products were separated through a 0.8% w/v agarose gel in TBE at 75 V for 65 min at 4°C, then transferred to nylon membrane (Hybond-N+) and UV-crosslinked (125 mJ for 80 s). Membranes were washed and blocked (Roche), and RNA probe was detected using anti-dig Fab fragment (Roche) and signal was revealed using 0.25 mM chemiluminescent substrate chloro-5- bysubstituted adamantly-1,2-dioxetane phosphate substrate (CSPD Roche). Quantification was carried out using FIJI, and data were expressed as percentages using the control lane of 0 μM protein as reference.

### Western blots

Samples were denatured in SDS–PAGE loading buffer at 90°C for 5–10 min, then separated through Mini-PROTEAN TGX precast gels 4%–15% (Bio-Rad), using running buffer (25 mM Tris, 192 mM Glycine, 0.1% SDS), and compared to PageRuler Plus Prestained Protein Ladder (Thermo Scientific). Separated proteins were transferred to nitrocellulose (GE Healthcare) using iBlot2 transfer system (Invitrogen). Membranes were blocked in antibody-appropriate buffers—5% BSA, 5%–10% non-fat dried milk diluted in phosphate or Tris buffered saline (PBS or TBS) with 0.1% Tween20—then incubated with primary antibodies (Table 3), washed in blocking buffer, then incubated with HRP-conjugated secondary antibodies. Signal was revealed using ECL chemiluminescence substrate (Thermo Scientific) and PXi gel imaging system (Syngene) with Genesys V1.8.2.0 software. Quantification of western blots was carried out using Gene Tools software 4.3.14.0 (Syngene). For visualization of protein content, gels were stained with Coomassie Blue (Invitrogen) and membranes with Ponceau S stain (Merck).

**Table 3.** Antibodies

Target	Source
CIZ1 (C-terminal tail) Rabbit anti-peptide	Novus (NB100-74624)
CIZ1 (N-term, 1794) Rabbit anti-recombinant CIZ1	[12]
CIZ1 (C-terminal tail) Goat anti-peptide	this study
CIZ1 (C-terminus, 87) Mouse anti-recombinant CIZ1	[9]
CIZ1 (MH3 domain)	Biorbyt (orb524815)
SAFA	Abcam (ab10297)
SAFA	Proteintech (14599-1-AP)
SAFB2	Proteintech (11642-1-AP)
SAFB2	Santa Cruz Biotechnology (sc-514963)
H2AK119ub1	Cell Signalling Technology (D27C4)
Tri-Methyl-Histone H3 (Lys27)	Cell Signalling Technology (C36B11)
α-Tubulin	Abcam (ab7291)
Aurora Kinase B (AURKB)	Novus (NBP2-50039)
SMARCA5	Biorbyt orb340809
NUMA1	Antibodies.com (A12645)
Histone H3	Abcam (Ab1791)
GFP	Sigma (SAB4301138)
Actin	Abcam (ab11003)
Actin	Sigma (A4700-2 mL)
GST	Abcam (ab9085)
Goat anti-rabbit IgG Alexa Fluor 568 (Red)	Invitrogen (A-11011)
Goat anti-mouse IgG Alexa Fluor 488 (Green)	Invitrogen (A-11001)
Goat anti-rabbit IgG Alexa Fluor 488	Invitrogen (A11034)
Goat anti-mouse IgG Alexa Fluor 568	Invitrogen (A11031)
Donkey anti-rabbit IgG Alexa Fluor 568 (Red)	Invitrogen (A-10042)
Donkey anti-goat IgG Alexa Fluor 488 (Green)	Invitrogen (A-11055)
HRP-IgG anti rabbit	Jackson Immuno (211-032-171)
HRP-IgG anti mouse	Jackson Immuno (155-035-174)

### Identification of interaction partners

Protein identification and relative quantification were carried out by label-free mass spectrometry, providing log_2_ protein abundance differences, and statistical significance between paired sample groups for all identified proteins.

Proteins were digested on-bead with sequencing-grade trypsin as described [15]. In study A, peptides were desalted with Millipore C_18_ ZipTip before being resuspended in aqueous 0.1% trifluoroacetic acid (v/v) and then loaded onto an mClass nanoflow UPLC system (Waters) equipped with a nanoEase M/Z Symmetry 100 Å C_18_, 5 µm trap column (180 µm × 20 mm, Waters) and a PepMap, 2 µm, 100 Å, C_18_ EasyNano nanocapillary column (75 μm × 500 mm, Thermo). The trap wash solvent was aqueous 0.05% (v:v) trifluoroacetic acid and the trapping flow rate was 15 µl/min. Separation used gradient elution of two solvents: solvent A, aqueous 0.1% (v:v) formic acid; solvent B, acetonitrile containing 0.1% (v:v) formic acid. The flow rate for the capillary column was 300 nl/min and the column temperature was 40°C. The linear multi-step gradient profile was 3%–10% B over 7 min, 10%–35% B over 30 min, and 35%–99% B over 5 min and then proceeded to wash with 99% solvent B for 4 min.

The nanoLC system was interfaced with an Orbitrap Fusion Tribrid mass spectrometer (Thermo) with an EasyNano ionization source (Thermo). Positive ESI-MS and MS^2^ spectra were acquired using Xcalibur software (version 4.0, Thermo). Instrument source settings were: ion spray voltage, 1900 V; sweep gas, 0 Arb; and ion transfer tube temperature, 275°C. MS^1^ spectra were acquired in the Orbitrap with 120 000 resolution, with a scan range of *m/z* 375–1500, AGC target of 4e^5^, max fill time, 100 ms. Data-dependent acquisition was performed in top-speed mode using a 1 s cycle, selecting the most intense precursors with charge states >1. Easy-IC was used for internal calibration. Dynamic exclusion was performed for 50 s post precursor selection and a minimum threshold for fragmentation was set at 5e^3^. MS^2^ spectra were acquired in the linear ion trap with: scan rate, turbo; quadrupole isolation, 1.6 *m/z*; activation type, HCD; activation energy: 32%; AGC target, 5e^3^; first mass, 110 *m/z*; max fill time, 100 ms. Acquisitions were arranged by Xcalibur to inject ions for all available parallelizable time.

Peak lists in .raw format were imported into Progenesis QI (version 2.2, Waters), and LC-MS runs were aligned. Precursor ion intensities were normalized against total intensity for each acquisition. A combined peak list was exported in .mgf format for database searching against the human subset of the SwissProt database (20 242 sequences; 11 289 677 residues), appended with common proteomic contaminants (116 sequences; 38 371 residues). Mascot Daemon (version 2.6.0, Matrix Science) was used to submit the search to a locally running copy of the Mascot program (Matrix Science Ltd., version 2.7.0). Search criteria specified: Enzyme, trypsin; Max missed cleavages, 1; Fixed modifications, Carbamidomethyl (C); Variable modifications, Oxidation (M); Peptide tolerance, 3 ppm; MS/MS tolerance, 0.5 Da; Instrument, ESI-TRAP. Peptide identifications were passed through the percolator algorithm to achieve a 1% false discovery rate assessed against a reverse database and individual matches were filtered to require minimum expected score of 0.05. The Mascot .XML result file was imported into Progenesis QI, and peptide identifications associated with precursor peak areas and matched between runs. Relative protein abundance was calculated using precursor ion areas from non-conflicting unique peptides. Accepted protein quantifications were set to require a minimum of two unique peptide sequences. Statistical testing was performed in Progenesis QI from ArcSinh-normalized peptide abundances and ANOVA-derived *P*-values were converted to multiple test-corrected *q*-values within Progenesis software.

For studies B and C, LC-MS acquisition was performed using a Bruker TimsTOF HT mass spectrometer. Peptides were loaded onto EvoTip Pure tips for nanoUPLC using an EvoSep One system. A pre-set 60 SPD gradient was used with an 8 cm EvoSep C_18_ Performance column (8 cm × 150 μm × 1.5 μm). The nanoUPLC system was interfaced to a timsTOF HT mass spectrometer (Bruker) with a CaptiveSpray ionization source (Source). Positive PASEF-DDA, ESI-MS, and MS^2^ spectra were acquired using Compass HyStar software (version 6.2, Bruker). Instrument source settings were: capillary voltage, 1600 V; dry gas, 3 l/min; and dry temperature; 180°C. Spectra were acquired between *m/z* 100 and 1700. TIMS settings were 1/K_0_ 0.6–1.60 V.s/cm^2^; ramp time, 100 ms; ramp rate, 9.42 Hz. Equipment is specified in Table 4. Data-dependent acquisition was performed with 10 PASEF ramps and a total cycle time of 1.17 s. An intensity threshold of 2500 and a target intensity of 20 000 were set with active exclusion applied for 0.4 min post precursor selection. Collision energy was interpolated between 20 eV at 0.6 V.s/cm^2^ and 59 eV at 1.6 V.s/cm^2^. Data in Bruker .d format were searched using FragPipe (v20.0) [16] against the human subset of UniProt appended with common proteomic contaminants. Search criteria were set as for study A, with the exception that mass tolerance was set to 15 ppm for both MS^1^ and MS^2^. Peptide identifications were processed using Philosopher (v5.0.0) to achieve a 1% false discovery rate as assessed against a reverse database. Relative protein quantification was extracted from precursor ion areas using IonQuant (v 1.9.8) with match between runs. Data were filtered to require a minimum of two unique peptides before applying sample minimum imputation. Pairwise statistical comparison was performed using limma [17] via FragPipe-Analyst [18]. *P*-values from pairwise testing were multiple-test corrected to *q*-value FDR using local and the Hochberg and Benjamini approach. If a protein was deemed to interact with CIZ1, its percentage contribution to total ion area had to be ≥2-fold higher than its value when retrieved by GST controls, with *q* ≤ 0.05 considered significant.

**Table 4.** Other resources

Reagent or resource	Source	Identifier
**Bacteria and vectors**		
*E. coli* DH5α Competent Cells	Invitrogen	Cat#18265017
*E. coli* BL21-CodonPlus Competent Cells	Agilent Technologies	Cat#230250
pCMV-Xist-PA	Addgene	Cat#26760
**Chemicals**		
Trypsin	Promega	V5111
Trypsin/Lys-C	Promega	V5071
Glu-C	Promega	V1651
TiO2	MagReSyn	MR-TID-002
High Glucose DMEM GlutaMAX	Gibco	Cat#531966-021
Pen/Strep/Glutamine	Gibco	Cat#10378016
Dulbecco’s PBS	Gibco	Cat#14190
Trypsin-EDTA (0.5%)	Gibco	Cat#15400-054
Opti-MEM	Gibco	Cat#31985-062
TransIT-X2	Mirus	Cat#MIR6003
Vectashield with DAPI	Vector Labs	Cat#H-1200-10
Nocodazole	Sigma	Cat#M1404
Thymidine	Sigma	Cat#T1895
Okadaic acid	CST	Cat#5934S
Tautomycin	Sigma	Cat#580551
Barasertib	Selleck	Cat#AZD1152-HQPA
CloneAmp HiFi PCR premix	Takara	Cat#639298
QIAprep Spin Miniprep Kit	QIAGEN	Cat#27104
QIAprep Spin Midiprep Kit	QIAGEN	Cat#12143
EZBlock Protease Inhibitor Cocktail, EDTA-Free	BioVision	Cat#K272-1
PreScission protease	Cytiva	Cat#27-0843-01
DIG Wash and Block Buffer Set	Roche	Cat#11585762001
Anti-Digoxigenin-AP, Fab fragments	Roche	Cat#11093274910
Digoxigenin-11-UTP	Roche	Cat#11209256910
chloro-5-substituted adamantly-1,2-dioxetane phosphate substrate (CSPD)	Roche	Cat#11655884001
MEGAshortscript™ T7 Transcription Kit	Ambion	Cat#AM1354
MEGAclear™ Transcription Clean-Up Kit	Invitrogen	Cat#AM1908
Glutathione Sepharose^®^ 4B 10 ml	Cytiva	Cat#GE17-0756-01
Benzonase	Millipore	Cat#70746-2.5KUN
RNase, DNase free	Roche	Cat#11119915001
SSC	SLS	Cat#NAT1224
RNase OUT	Invitrogen	Cat#10777019
PageRuler Plus Prestained Protein Ladder	Thermo scientific	Cat#26619
Mini-PROTEAN TGX precast gels	Bio-Rad	Cat#456-1085
Nitrocellulose membrane	GE Healthcare	Cat#10600000
ECL chemiluminescence substrate	ThermoScientific	Cat#32109
Coomassie stain	Invitrogen	Cat#LC6065
Ponceau S stain	Merck	Cat#P7170-1L
DpnI	NEB	Cat#R0176
LB agar	Millipore	Cat#110283
**Protein expression constructs**		
GST-C179_WT_ (Human CIZ1)	This study	N/A
GST-C179_Δtail_ (Human CIZ1)	This study	N/A
GST-C179_DDD_ (Human CIZ1)	This study	N/A
GST-C181_WT_ (Murine CIZ1)	[9]	N/A
GST-C181_ΔMH3_ (Murine CIZ1)	This study	N/A
GST-C181_Δtail_ (Murine CIZ1)	This study	N/A
GST-C181_ΔAcD_ (Murine CIZ1)	This study	N/A
GST-C181_ΔMH3Δtail_ (Murine CIZ1)	This study	N/A
GST-C181_ΔAcDΔMH3_ (Murine CIZ1)	This study	N/A
Aurora kinase B	Abcam	Cat#ab51435
Aurora kinase B	Merck/Millipore	Cat#14-835
**Transfection constructs**		
GFP-m845_WT_	[12]	N/A
GFP-m845_Δ15_	This study	N/A
GFP-m845_AAA_	This study	N/A
GFP-m845_DDD_	This study	N/A
GFP-C275_WT_	[4]	N/A
GFP-C275_DDD_	This study	N/A
**Equipment**		
GSTrap 4B column	Cytiva	Cat#28401747
Superdex^®^ 200 Increase 10/300 GL column	Cytiva	Cat#28-9909-44
Superdex® S200 10/300 GL column	GE Healthcare	Cat#10245604
Millipore C18 ZipTip	Merck Millipore	ZTC18M096
nanoEase M/Z Symmetry C18 Trap Column	Waters	186008821
Performance C18 Nano column	EvoSep	EV1115
EvoTip Pure loading tips	EvoSep	EV2015
C18 EasyNano nanocapillary column	Thermo	ES803A
S-Trap Micro Column	Profiti	C02-micro-80
**Software and website**		
UNICORN^™^ ÄKTA control software	Cytiva	N/A
PXi GenSys software	Syngene	N/A
Axiovision image acquisition software (SE64 release 4.9.1)	Zeiss	N/A
FIJI	[19]	https://imagej.net/
STRING	[20]	https://string-db.org/
Group based prediction system	[21]	https://gps.biocuckoo.cn/citation.php
cytoscape 3.10.2.	[22]	https://cytoscape.org/
Jalview 2.11.3.	[23]	https://www.jalview.org/
Livecyte 2	Phasefocus	https://www.phasefocus.com/livecyte
limma	[17]	https://bioconductor.org/packages/release/bioc/html/limma.html
Orbitrap software, Xcalibur v4.0	Thermo	
TimsTOF HT control software, Compass HyStar v 6.2	Bruker	
Progenesis QI v2.2	Waters	
Mascot Daemon v2.6.0	Matrix Science	
Mascot Server v2.7.0	Matrix Science	
FragPipe (v20)	[16]	https://fragpipe.nesvilab.org/
Philosopher (v5.0.0):	[24]	https://philosopher.nesvilab.org/
IonQuant (v 1.9.8):	[25]	https://msfragger-upgrader.nesvilab.org/ionquant/
FragPipe-Analyst	[18]	https://fragpipe-analyst.org/

### *In vitro* phosphorylation

Reactions analysed by western blot were set up using 62.5 ng of recombinant C179_WT_ as substrate with a titration of 0–400 ng Aurora Kinase B (Abcam, ab51435) in 20 mM HEPES pH 7.5, 5 mM MgCl2, 0.1 mM ATP, 1 mM ATP (20 microlitres final volume), incubated at 30°C for 30 mins, and stopped by addition of SDS–PAGE loading buffer. Reactions analysed by mass spectrometry contained 5.43 μg recombinant C179_WT_ and 3.15 µg Aurora Kinase B (Merck/Millipore, 14-835), or no kinase control, in 8 mM MOPS/NaOH pH 7.0, 0.2 mM EDTA, 1% glycerol, 0.02% v/v β-mercaptoethanol, 10 mM MgCl_2_, 0.1 mM ATP, and were incubated at 30°C for 30 min. Five micrograms of kinase-treated and untreated C179_WT_ were split and digested in parallel with either trypsin or Glu-C sequencing-grade proteases using an S-Trap (Micro Column, Profiti)-mediated procedure. A 90% aliquot of digested material was enriched for phosphopeptides using MagReSyn titanium dioxide beads as detailed by manufacturer. LC-MS data were acquired for both enriched and non-enriched peptides for interaction studies B and C, with the exception that a 100 SPD EvoSep gradient was used. Spectra were searched using FragPipe with Glu-C specificity included as applicable and variable phosphorylation of S, T, and Y residues was considered. Searches were run with a 1% FDR cut-off. Phosphorylation site localization confidence was extracted using Philosopher (v5.0.0). CIZ1 phosphopeptide identifications were combined for all searches.

### Bioinformatics

Putative kinase sites were mapped on human and murine CIZ1 using Group-based Prediction Software GPS 5.0 [21], with additional consensus for AURKB sites from [26]. Analysis of CIZ1 interaction partners was carried out using the STRING database. Networks applied MCL clustering (Markov cluster algorithm) and an inflation parameter of 3, displaying evidence from experimentally determined sources, curated databases, and co-expression studies. Outputs were visualized using Cytoscape 3.10.2 [22].

### Cloning

Site-directed mutagenesis was carried out by PCR, using primers listed in Table 2. The products were digested with 2U DpnI (NEB) diluted in 1× CutSmart buffer (NEB), then transformed into DH5α cell suspension (Invitrogen) and grown at 37°C overnight with antibiotic selection. Single colonies were chosen to inoculate 5 ml LB broth for overnight growth and used for plasmid purification via the QIAprep Spin Miniprep Kit (QIAGEN). Sequences were verified by Eurofins TubeSeq service. Plasmids used for protein expression in *E. coli* were retransformed into BL21 RP cells (Agilent). GFP-m845_Δ15_ (also known as GFP-m830), lacking the terminal 15 amino acids, was generated by deletion of a 260 bp BamH1 fragment from the C-terminal end of GFP-m845 [12] followed by relegation.

## Results

### Phosphorylation-regulated dissolution of CIZ1–*Xist* assemblies during the cell cycle

During interphase the CIZ1 assemblies that aggregate around Xi chromatin are readily visualizsed by immunofluorescence microscopy in murine fibroblasts and other female mouse and human cell types [5–7, 10, 27, 28]. However, they become undetectable during the metaphase-to-anaphase transition and detectable again ~4 h into G1 phase (Fig. 1B and C) [10]. This mirrors the cyclical behaviour of *Xist* [29], which is reported to be regulated by the serine/threonine mitotic protein kinase Aurora B (AURKB) [30]. Human and murine CIZ1 each encode six canonical AURKB phosphorylation sites, of which five are conserved (Supplementary Fig. S1A): one in the N-terminal half of CIZ1, another in the second zinc finger, and a cluster of three at the extreme C-terminus (Fig. 1D). The C-terminal 38 amino acids of CIZ1 are predicted to be unstructured in both human and mouse, which possess the same conserved domains (Supplementary Fig. S1B), encoded by the same exons in the same order. This unstructured tail domain and its conserved AURKB site cluster are functionally unexplored.

Initial experiments to explore the cyclical behaviour of CIZ1–Xi assemblies quantified the effect of the AURKB inhibitor barasertib [11] during passage through mitosis. In both D3T3 cells and female primary embryonic fibroblast populations (PEFs at passage 3), the frequency of cells with CIZ1 assemblies remained stable in interphase and prophase (93%–94%), dropped to ~60% in metaphase, and was almost absent by anaphase (Fig. 1E). However, in the presence of barasertib, we observed a dose-dependent retention of CIZ1–Xi assemblies in both types of cell in anaphase, increasing from 3% in untreated cells to 84% in the presence of 1 µM barasertib. Consistent with the conclusion that phosphorylation promotes dissolution of CIZ1–Xi assemblies, both the broad-spectrum protein phosphatase inhibitor okadaic acid [31] and the concentration-dependent protein phosphatase 1/2A inhibitor tautomycin [32] shifted the regulatory axis and caused dispersal of CIZ1–Xi assemblies in interphase cells within hours (Fig. 1F). Dispersal was observed when detected via an epitope in the N-terminal half of CIZ1, and a more complete loss when detected via an epitope in the C-terminal tail of CIZ1 (Fig. 1F). Previous work indicates that CIZ1 and *Xist* coexist within ribonucleoprotein particles, and that their accumulation around Xi is co-dependent in fibroblasts [5, 6]. Consistent with this, *Xist* was also dispersed from interphase cells upon phosphatase inhibition (Supplementary Fig. S1D), as reported previously [30]. Together the data argue that release of CIZ1 and *Xist* from Xi is co-regulated by cycles of phosphorylation that involve AURKB. The data also suggest that during interphase either the association between CIZ1 and *Xist* or the aggregation of CIZ1–*Xist*-containing RNPs is actively protected by the action of a phosphatase.

### Direct or indirect effect?

AURKB may phosphorylate CIZ1 to modulate its relationship with *Xist*; however, other *Xist*-interaction partners are similarly regulated, and so effects could be indirect. Notably, the DNA-binding domain of HNRNPU/SAFA is phosphorylated by AURKB and is associated with release of chromosome-associated RNAs during chromosome segregation [33]. SAFA normally tethers chromosomal RNAs to chromatin throughout the nucleus in interphase [34], via direct interaction with AT-rich S/MARs [35], and has long been implicated in retention of *Xist* within Xi chromatin territories [36]. In our experiments SAFA is lost from the nucleus earlier in mitosis than CIZ1–Xi assemblies and was also (partially) protected by barasertib in prophase (Fig. 1E). Thus, while AURKB could act directly on CIZ1 to drive its disassembly, these data do not rule out an indirect effect in which phosphorylation of SAFA leads to dispersal of CIZ1 and *Xist*.

### Disruption of the C-terminal tail perturbs nucleus-wide anchoring of CIZ1

**Figure 2.**
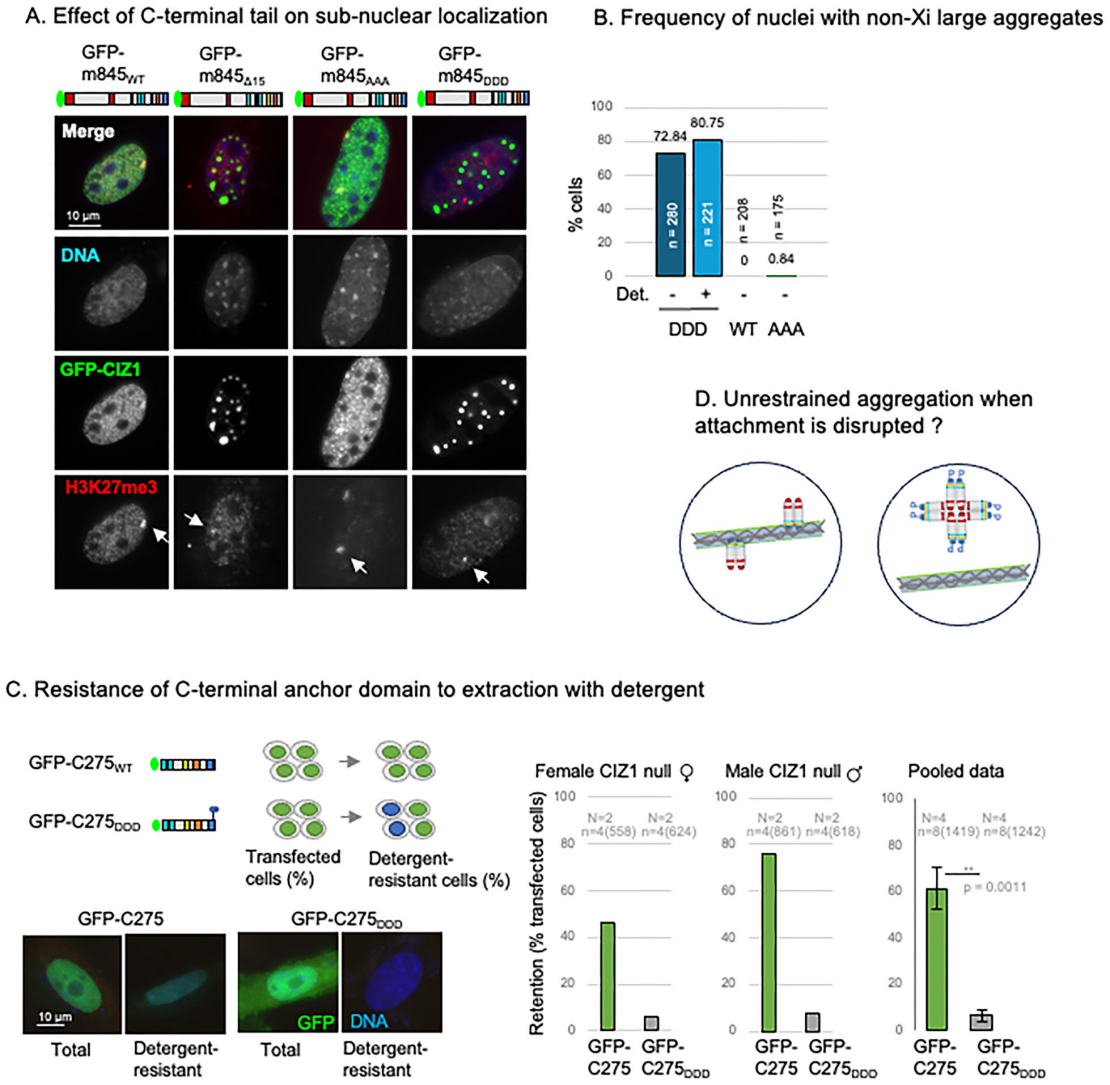
The C-terminal tail specifies nuclear immobilization and limits aggregation. (**A**) Immunofluorescence images showing female murine fibroblasts transfected with full-length mouse GFP-845, or derived constructs m845_Δ15_ (lacking the C-terminal 15 amino acids), m845_DDD_ or m845_AAA_ (green). Cells were co-stained for H3K27me3 (red). DNA is shown in blue, bar is 10 microns. (**B**) Frequency of nuclei containing large (non-Xi) CIZ1 aggregates, shown as percentage of cells transfected. Results are shown with (+) and without (−) prefixation detergent wash (Det.). *n* = transfected nuclei counted. (**C**) Schematic showing the C-terminal portion of CIZ1, GFP-mC275 and derived GFP-mC275 _DDD_, and their use in 48 h transient expression experiments to assess their ability to assemble into detergent-resistant structures [4]. Below, example images of transfected nuclei. Histograms show retention frequency in male (N = 2) and female (N = 2) CIZ1 null primary embryonic fibroblasts that were transfected (green). *n* denotes technical replicates for each cell population, with number of nuclei scored in parentheses. (**D**) Illustration displaying the effect of AURKB site cluster phosphomimic (P) on CIZ1’s association with chromatin and associated detergent-resistant nuclear structures.

To test whether the cluster of three putative AURKB sites in the extreme C-terminus of CIZ1 are functionally relevant, we generated a murine GFP–CIZ1 fusion bearing phosphomimetic aspartic acid substitutions in all three sites at positions 840, 841, and 845 (Fig. 1D) to create GFP-m845_DDD_. When transfected into mammalian cells GFP-m845_WT_ assumes a similar sub-nuclear localization pattern to the endogenous protein accumulating at both the Xi (in females) and at smaller foci dispersed across the nucleus (in both sexes) (Fig. 2A). This requires both N- and C-terminal domains of CIZ1, is dependent on low-complexity prion-like domains that support self-aggregation, and can occur against a CIZ1 null background [9]. However, both GFP-m845_DDD_ and a deletion mutant lacking the terminal 15 amino acids, including the cluster of AURKB sites (GFP-m845_Δ15_), displayed a dramatic phenotype of large subnuclear aggregates (Fig. 2A and B). In contrast, conversion of the AURKB site cluster to an unphosphorylatable state (GFP-CIZ1_AAA_) did not affect its localization. Thus, the phosphomimetic mutations drive a dramatic loss of normal function within the C-terminal tail that affects spatial pattern.

When tested in the context of the C-terminal nuclear matrix anchor domain alone (GFP-C275), which lacks the prion-like domains that drive coalescence, GFP-C275_DDD_ revealed failure to become immobilized by attachment to insoluble nuclear structures. Like GFP-C275 [4, 9], GFP-C275_DDD_ was expressed and imported into the nucleus (where it did not form large aggregates), but its resistance to extraction by detergent was reduced from 61.3% resistant nuclei to 6.6%. This was tested in CIZ1 null cells to avoid complications arising from possible association with the endogenous protein, and in both male and female cells to enable wider interpretation of the results beyond possible interaction with female-specific *Xist* (Fig. 2C). Together these observations suggest the C-terminal tail supports anchorage of CIZ1 in the nucleus and are also consistent with the idea that this limits the PLD-driven tendency of CIZ1 to self-aggregate into large assemblies (Fig. 2D).

### Modification of C-terminal AURKB sites in metaphase

**Figure 3.**
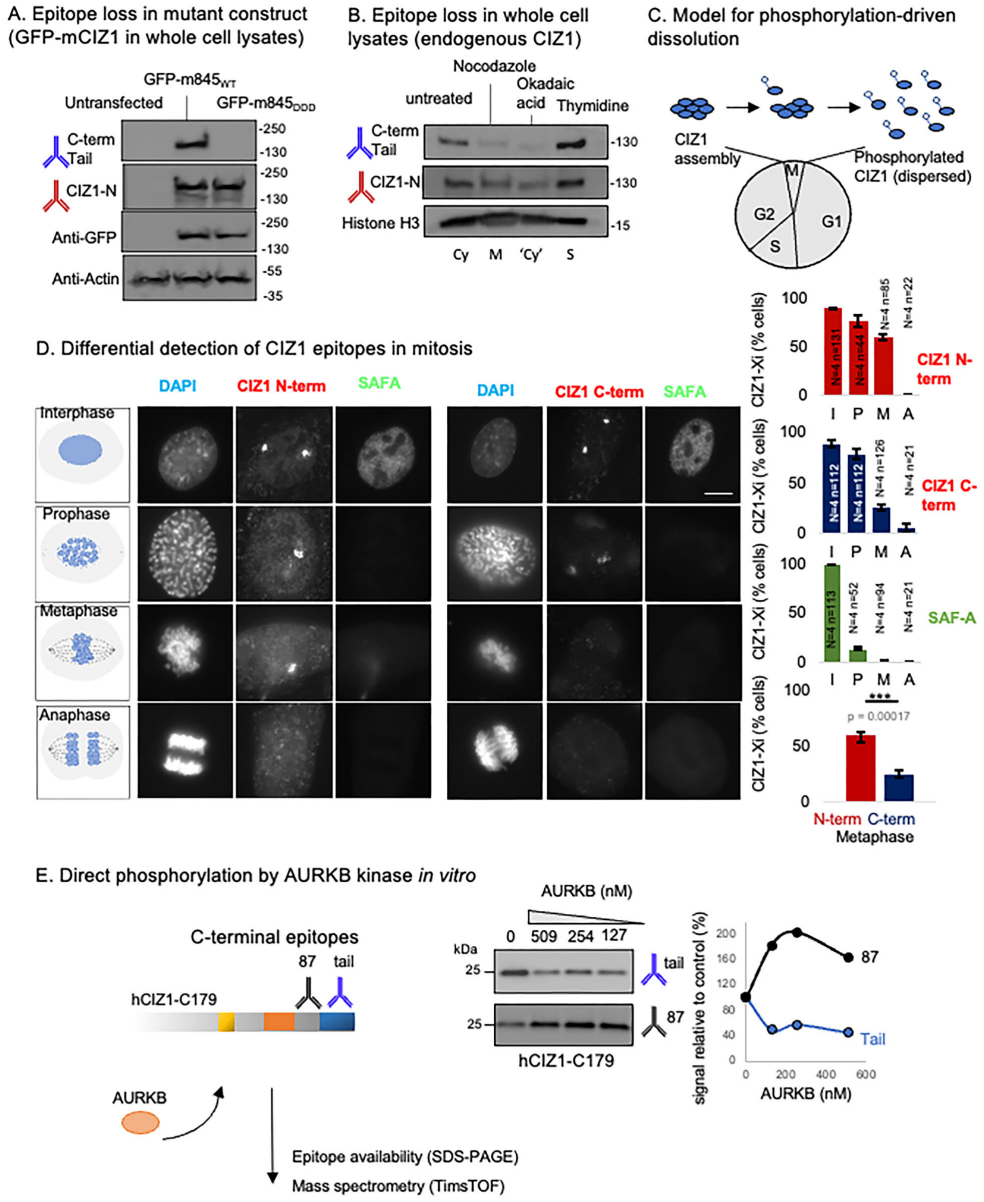
AURKB site modification in mitosis. (**A**) Western blot showing denatured proteins in whole cell lysates collected from untransfected D3T3 cells, or populations expressing full-length mouse GFP-m845_WT_ or GFP-m845_DDD_, after immunostaining for CIZ1-N or CIZ1-C (tail epitope), or β-actin, and GFP as indicated. (**B**) Western blot showing denatured endogenous proteins in chemically treated D3T3 cells to achieve cell cycle enrichment in mitosis (M, nocodazole), S phase (S, thymidine), or a phosphatase-suppressed state (okadaic acid). Immunoblotting for C-terminal tail and N-terminal CIZ1 indicates a reduction in tail epitope, compared to untreated cells, during arrest in metaphase, or after phosphatase inhibition. Histone H3 is shown as a loading control. Cy, cycling. (**C**) Illustration showing data interpretation in which the CIZ1 tail AURKB site cluster is phosphorylated in mitosis, driving dispersal of CIZ1 from Xi assemblies. (**D**) Female D3T3 cells in stages of mitosis as indicated, immunostained for CIZ1-N or CIZ1-C and co-stained for SAFA. Right, histograms show frequency of retention in interphase (I), prophase (P), metaphase (M), or anaphase (A), where N indicates replicate analyses and n nuclei scored. Lower, by metaphase CIZ1-N and CIZ1-C are significantly different (*P *< .00017), student’s t-test. (**E**) Experimental overview of *in vitro* kinase reactions using purified recombinant human CIZ1 C-terminal fragment C179 and purified AURKB. Middle, products analysed by western blot with C-terminal CIZ1 epitope-defined antibodies, showing changes in reactivity in response to exposure to increasing concentrations of AURKB kinase. Graph shows band intensities relative to untreated C179 control. Products were also analysed by mass spectrometry (Supplementary Fig. S2C).

Anti-CIZ1 peptide antibody, raised against sequences in the C-terminal tail in which the AURKB sites reside (Fig. 1D and Supplementary Fig. S1C, ‘tail antibody’), does not detect ectopic full-length GFP-m845_DDD_ in cell lysates by western blot, while an N-terminal anti-CIZ1 antibody does (Fig. 3A). This shows that its epitope is affected by the mutations. When applied to whole cell lysates from synchronized cells expressing only endogenous CIZ1, it also strikingly fails to robustly detect the C-terminal epitope in some contexts. Cell populations arrested in mitosis, or cycling populations treated with the phosphatase inhibitor okadaic acid, were poorly detected compared to N-terminal epitopes (Fig. 3B), and compared to untreated cycling populations or those arrested in S phase, in which N- and C-terminal epitopes are both available. This suggests that CIZ1 is modified by phosphorylation of C-terminal tail sequences in mitosis (Fig. 3C), and that the event can be reported via this phospho-sensitive anti-tail antibody.

N-terminal and C-terminal antibodies also report different dynamics during mitosis when used for immunostaining of endogenous CIZ1 (Fig. 3D). Though the frequency of cells with CIZ1-marked Xi’s is similar in interphase populations (88.5% C-term, 89.1% N-term) and prophase populations (78.1% C-term, 75.8% N-term), in metaphase nuclei the C-terminal epitope is significantly less frequent (24.6% C-term, 58.8% N-term). This was confirmed using a different (goat) anti-tail antibody in co-staining experiments (Supplementary Fig. S2A) and suggests that the tail epitope is modified before assembly dissolution. Together the data support the idea that CIZ1 is normally phosphorylated in its C-terminal AURKB sites between prophase and metaphase, likely contributing to dissolution of CIZ1–Xi assemblies.

### AURKB directly phosphorylates the CIZ1 C-terminus

To confirm that CIZ1 can be directly phosphorylated by AURKB, recombinant human CIZ1-C179 protein was incubated with recombinant AURKB, and the impact was assessed using phosphosensitive C-terminal tail antibody by western blot (Fig. 3E). Compared to mock reactions, AURKB reduced the reactivity of the C-terminal tail antibody by 54%, supporting direct modification of the tail. Interestingly, AURKB exposure also increased accessibility to the C-terminal mAb 87 epitope by 63%, despite its epitope being located upstream of the putative kinase sites in the linear protein sequence (Fig. 1D). The products of AURKB exposure were further probed in a phosphoproteomic analysis, which identified 10 high-confidence targets in recombinant hC179 after exposure to AURKB, six within the C-terminal tail (Supplementary Fig. S2B and C). Of the three clustered conserved AURKB sites that were mutated only the terminal residue (Thr 898) was among the high-confidence sites. Nevertheless, these data confirm that CIZ1 is a potential substrate of AURKB.

### C-terminal interaction partners are enriched in chromatin and nuclear matrix proteins

**Figure 4.**
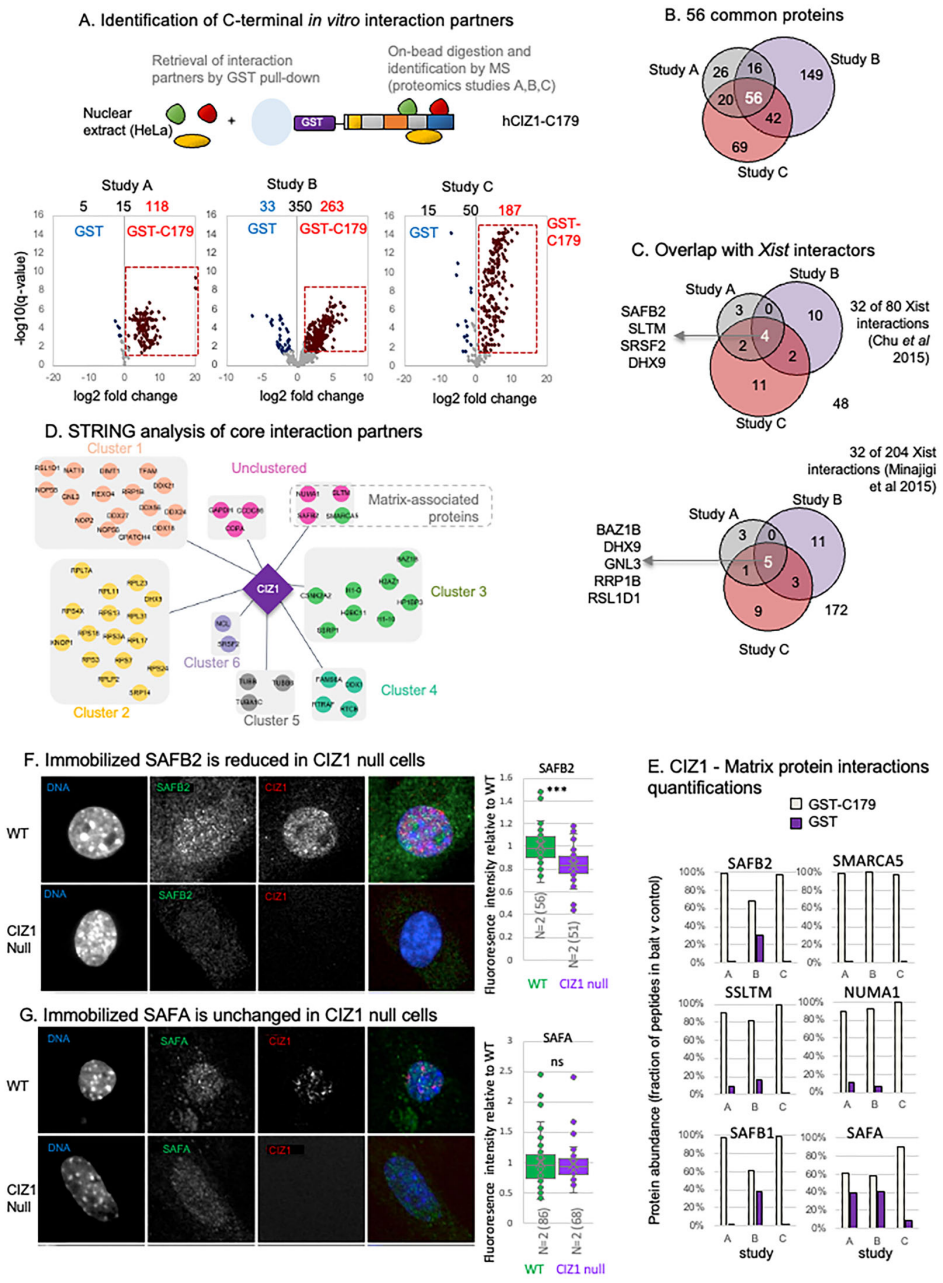
CIZ1 C-terminal interaction partners. (**A**) Overview of nuclear protein interaction studies using mammalian cell nuclear extracts from HeLa cells and recombinant GST-tagged hCIZ1 C-terminal fragment C179. Domains coloured as in Fig. 1D. Below, volcano plots showing protein interaction partners identified with high confidence in three independent studies and their relative retrieval by GST-hC179 compared to GST control. Significance (−log_10_ FDR *q*-value) is plotted against log_2_ fold change (FC), derived from *n* = 4 replicates in each case. Significant interaction partners are ≥2-fold more abundant in CIZ1–retrieved samples compared to GST samples, at *q*-value ≤0.05. Data are given in Supplemental Dataset 1. (**B**) Venn diagram showing common interaction partners between three independent studies. The 56 core interaction partners are listed in Supplemental Dataset 2. (**C**) CIZ1 interaction partners in common with 80 *Xist* interactors identified by CHIRP-MS [37] or iDRIP [38]. Venn diagram indicates those that interact with CIZ1 in all three of our studies. Proteins that were common to both *Xist* studies and also identified in any of our CIZ1 interaction studies are listed in Supplemental Dataset 2. (**D**) Simplified STRING diagram showing 56 core CIZ1 interaction partners, clustered using MCL clustering. Three unclustered proteins (dark pink) and one chromatin protein (green) are nuclear matrix-associated proteins (Supplemental Dataset 2). (**E**) Individual abundance (mean of four replicates) across three studies for four nuclear matrix proteins in the core 56 interaction list (SAFB2, SMARCA5, NUMA1, SLTM) and two that appear in at least one of the studies (SAFA, SAFB1). Histograms show fraction of high-confidence peptides in bait and control for each study. (**F**) Example immunofluorescence images of SAFB2 (green) in cycling WT and CIZ1 null PEFs, co-stained for CIZ1-N (red), and DAPI (blue). Box and whisker plots show intensity measures derived from two independent primary cell populations (N) for each genotype. *n* = number of nuclei measured. Comparison is by t-test, where *** denotes *P *< .001 and indicates a significant reduction of bound SAFB2 epitope in CIZ1 null cells. (**G**) As in panel (F) but for SAFA, which is not significantly changed in CIZ1 null cells.

To explore the interactions that normally anchor CIZ1 within the nucleus and which may be interrupted by phosphorylation of the AURKB site cluster in the CIZ1 tail, we first established a protein interaction network. Three independent studies, using the C-terminal 179 amino acids of human CIZ1 with an N-terminal GST tag, retrieved high-confidence interaction partners from nuclear extracts prepared from cycling HeLa cells (Supplemental Dataset 1). Across the three studies, 118, 263, and 187 proteins were identified that prefer GST-hC179 compared to GST alone (Fig. 4A), of which 56 were identified in all three studies, and a further 78 in two of the three studies (Fig. 4B and Supplemental Dataset 2).

*Xist* lncRNA has been extensively probed to identify protein interaction partners in different cell types, using a range of methods [37–41]. We compared our core list of 56 human CIZ1 interaction partners to the 81 proteins retrieved from four stem cell lines by *Xist* CHIRP-MS [37] and the 204 identified as direct *Xist* interacting proteins by iDRiP in embryonic fibroblasts [38]. Excluding CIZ1 itself, four proteins (DHX9, SAFB2, SLTM, SRSF2) and five proteins (BAZ1B, DHX9, GNL3, RRP1B, RSL1D1) were common across all three of our studies, but we observed a total of 32 different proteins in each case (Fig. 4C and Supplemental Dataset 2), including the nuclear matrix proteins SAFA, matrin-3, and HNRNPK [37].

STRING network analysis [20] of the 56 common proteins separated 50 into six clusters (Fig. 4D), and Gene Set Enrichment Analysis (GSEA) within the STRING platform assigned functional groups (Supplemental Dataset 2). Clusters 1 and 2 contain proteins linked with RNA binding and RNA processing, and include DHX9, which was previously reported to interact with the N-terminal part of CIZ1 [42], plus five other DEAD-box helicases. All CIZ1 interaction partners in cluster 3 belong to the cellular component chromatin (GO:0000785), including two nucleosomal histone variants and two variants of linker histone H1, as well as several nucleosome remodelling factors. The six unclustered proteins returned no GO processes or function; however, they include three nuclear matrix-associated proteins—NUMA1 [43] and the Scaffold attachment factor B family members SAFB2 and SLTM (SAFB-like transcription modulator) [44, 45]. SAFB2 was previously identified in the bioplex network as a potential CIZ1 interaction partner [46], and SAFB1 was significantly retrieved in two of the three interaction studies. SMARCA5, a component of the ‘Imitation SWItch’ (ISWI) chromatin remodelling complex, assigned to cluster 3 is also a nuclear matrix-associated protein (Fig. 4D and E).

To probe possible functional relationships between CIZ1 and this class of proteins, we looked at their subnuclear location and retention in CIZ1-null PEFs. Despite no significant difference in transcript levels compared to WT PEFs (Supplementary Fig. 3A), significant differences in their nuclear retention were observed for some. In interphase cells, SAFB2 is reduced, SAFA is unchanged, and SMARCA5 is increased (Fig. 4F and G, Supplementary Fig. 3B). Thus, SAFB2 may, in part, be dependent on CIZ1 for accurate assembly.

### CIZ1 null cells display segregation defects

In the same primary cell types, we observed CIZ1-related mitotic deficiencies. Two independent CIZ1 null PEF populations showed increased frequency of spindle abnormalities compared to WT (Supplementary Fig. S3C) and a significant increase in the frequency of aborted mitoses, as revealed by live cell tracking of parallel populations (Supplementary Fig. S3D–F). While the requirement for CIZ1 for high-fidelity spindle function is not yet understood, this does implicate it, and possibly its binding partners, in the accurate execution of mitosis.

### Phosphomimetic CIZ1 mutants have impaired interaction with RNA-binding proteins

**Figure 5.**
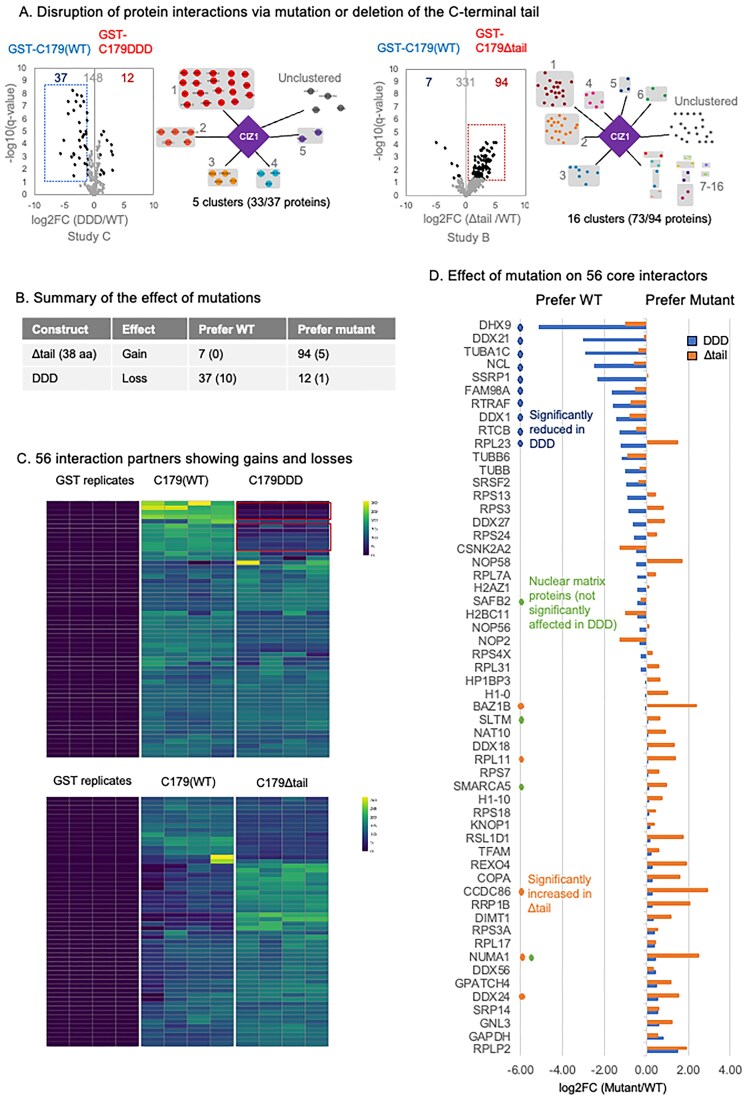
Effect of mutation on CIZ1 C-terminal interaction partners. (**A**) Volcano plots displaying protein interaction partners, comparing WT human C179 with C179_DDD_ or C179_Δtail_ in independent studies. The majority of proteins were unaffected. Those significantly increased or decreased are highlighted (log_2_ fold change ±1, *q* ≤ 0.05). See also Supplemental Dataset 2. To the right, STRING diagrams showing functional protein clusters for differentially retrieved proteins. Cluster identities are given in Supplemental Dataset 2. (**B**) Summary table describing effects of mutations on study-specific interaction partners and core 56 interaction partners (in parentheses). (**C**) Heatmaps showing peptide abundance of the core 56 interaction partners retrieved in control (GST), WT (C179), and mutant reactions, as indicated (*n* = 4 replicates). Upper, 10 proteins that are significantly reduced in C179_DDD_ compared to WT are indicated. Lower, of the core 56 only 5 are significantly changed [see panel (D)]. (**D**) Plot displays log_2_FC of core 56 proteins for C179_DDD_ (blue) and C179 _Δtail_ (orange), with identities. Ten proteins that are significantly reduced upon phosphomimetic mutation are labelled in blue. Nuclear matrix proteins SAFB2, SLTM, and SMARCA5 are not affected; however, NUMA1 was increased in C179 _Δtail_ (green).

The nuclear matrix proteins with which CIZ1 interacts are candidate factors that could support its localization and anchorage within the nucleus, and that may be sensitive to AURKB phosphorylation during CIZ1–Xi assembly dissolution in mitosis. We tested this by evaluating the effect of deleting the C-terminal 38 amino acids to create C179_Δtail_ or of converting the three clustered AURKB sites to aspartic acid to create C179_DDD._ Their ability to retrieve interacting proteins was compared to WT C179, as part of studies B or C, respectively, revealing unexpected results (Fig. 5A and B detailed in Supplemental dataset 2). Note that while the DDD mutation is identical to that used in the context of full-length CIZ1 in Fig. 2A, the _Δtail_ mutation excludes 38 amino acids rather than 15 in the _Δ15_ mutant.

While C179_DDD_ lost 37 proteins (including 10 of the core 56 interaction partners), complete deletion of the tail region resulted in no loss of any core proteins that met the significance threshold (FDR *q* < 0.05, log_2_ fold change >1). In fact, many are increased, including five core interaction partners (BAZ1B, CCDC86, DDX24, NUMA1, and RPL11), which contribute to a total of 94 proteins that prefer C179_Δtail_ compared to WT. One potential explanation for the emergence of new interaction partners is that the 38 amino acids that make up the unstructured tail region could block interaction sites located elsewhere.

Of the four nuclear matrix proteins in the core 56 interaction partners, none were lost (Fig. 5C and D). Thus, regulated dissolution of CIZ1 assemblies via phosphorylation of the C-terminal AURKB site cluster does not appear to be achieved via dissociation from other nuclear matrix proteins.

GSEA of the 37 proteins whose binding is significantly diminished upon phosphomimetic mutation revealed that 33 are associated with Gene Ontology (GO) molecular function RNA binding (GO:0003723, FDR 1.39e^−27^), of which 17 have RNA splicing function (GO:0008380, FDR 1.15e^−15^), while the 10 that are part of the core 56 interaction list include the entirety of cluster 4 tRNA-splicing ligase complex (Fig. 5A and D and Supplemental dataset 2). Thus, phosphomimetic mutation of CIZ1 does affect *in vitro* protein interactions, but 89% of those affected are themselves RNA-binding proteins. These could undergo direct protein–protein interaction with CIZ1 or could have been retrieved via indirect interaction mediated by RNA molecules present in the nuclear extract. Loss of RNA binding proteins leads us to ask whether phosphorylation of the C-terminal AURKB site cluster could cause dissolution of CIZ1–Xi assemblies by driving dissociation from direct interaction with RNA.

### CIZ1 exists as a stable dimer with MH3 domain interface

**Figure 6.**
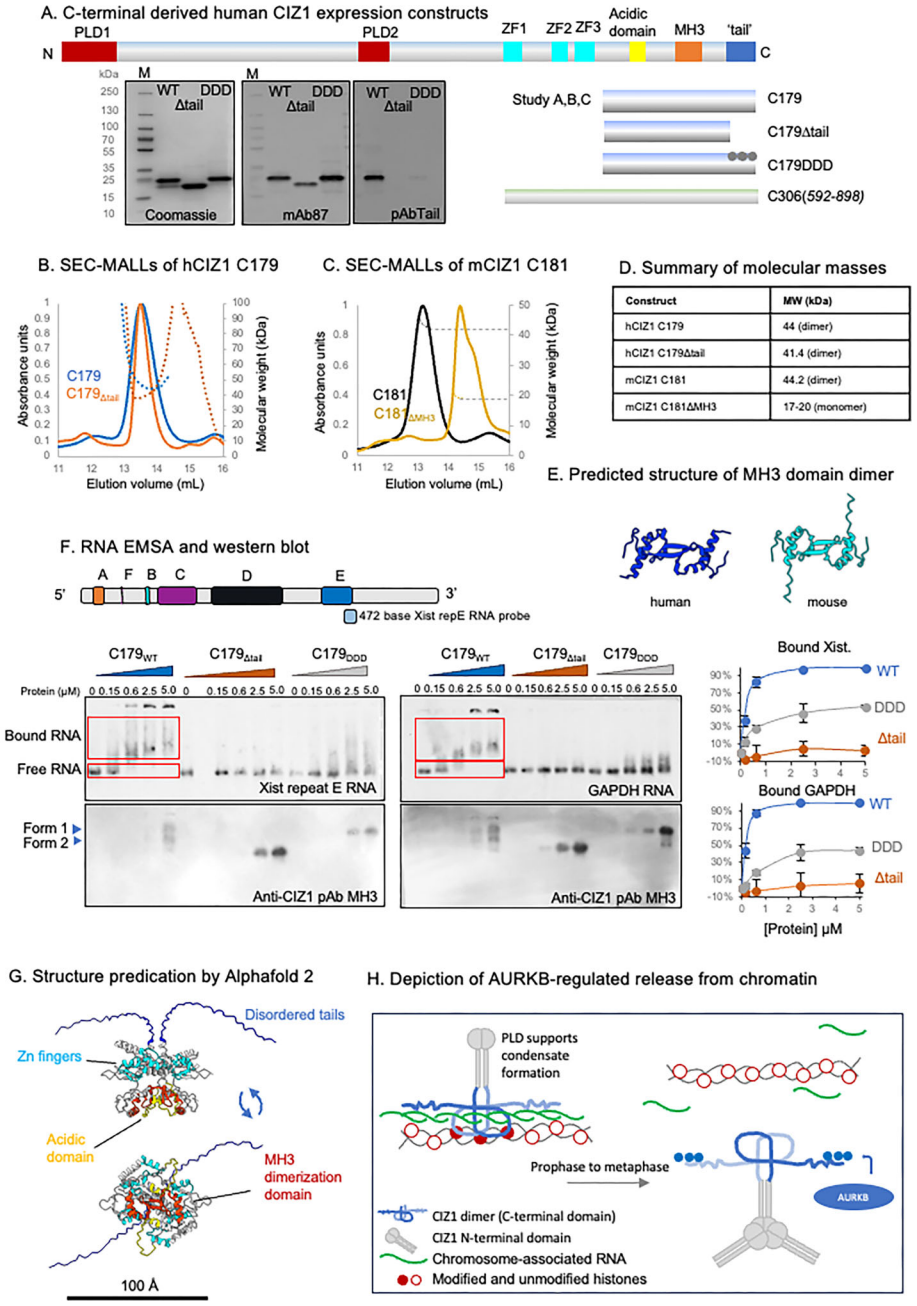
Interaction between CIZ1 dimer and RNA is regulated by AURKB sites in the C-terminal tail. (**A**) Schematic of h/m CIZ1 showing conserved domains, in yellow (acidic domain), orange (MH3 dimerization domain), and blue (unstructured tail h37/m38 C-terminal amino acids). Below, human C-terminal (C179) fragments, and derived mutants used as bait fragments in interaction studies, including C179_Δtail_ and phosphomimic C179_DDD_. Below, C-terminal fragment encompassing the Zn finger motifs used for modelling (green, C305). Left, SDS–PAGE gels showing purified protein preparations stained with Coomassie Blue, or probed with anti-CIZ1 mAb 87, which recognizes all three proteins, or anti-CIZ1 tail pAb, which recognizes an epitope deleted in C179_Δtail_ and mutated in C179_DDD_. (**B**) SEC-MALLS, showing normalized UV absorbance at 280 nm and molar mass (dotted line) for human CIZ1-C179 (blue), and human CIZ1-C179_Δtail_ (orange). (**C**) SEC-MALLS chromatogram showing normalized UV absorbance at 280 nm and molar mass (dotted line) for equivalent murine fragment C181 (black), and derived deletion mutant lacking the matrin 3 homology domain (C181_ΔMH3_, yellow). (**D**) Summary of measured molecular masses, indicating that the C-terminal fragment forms a stable dimer that is dependent on the MH3 domain but not the tail region. (**E**) AlphaFold dimer structure predictions of MH3 domain, showing human CIZ1 aa 779–838 uniprot Q9ULV3-1 (blue) and murine CIZ1 aa 725–785 uniprot Q8VEH2 (cyan). The domain forms a tight dimer with monomer–monomer interactions involving main chain hydrogen bonding between β-strands of the two MH3-type Zn finger motifs. (**F**) Example electrophoretic mobility shift assays (EMSA) showing the effect of C179, C179_Δtail_, and C179_DDD_ on the mobility of digoxygenin (DIG)-labelled *Xist* repeat E RNA probe (left, 0.66 nM) or GAPDH RNA (right, 0.65 nM). Below, immunoblots of EMSA membranes using CIZ1 anti-MH3 domain antibody. Above, murine *Xist* structure [49] and the derived *Xist* repeat E RNA probe used in EMSAs. Right, quantification of binding based on the fraction of shifted probe, derived from three replicate experiments (see also Supplementary Fig. S4). Graphs show means ± SEM. (**G**) AlphaFold-Multimer [47, 48] structure prediction of human C-terminal aa 592–898 (hC306), showing the highest-ranking prediction, in which the acidic domains (yellow) are exposed and the unstructured tails (blue) extend from the core. (**H**) Model, depicting CIZ1 homodimers interacting with chromosome-associated RNAs via its C-terminal tails, with N-terminal PLD domains available for association with other proteins or other RNAs (left). Right, shows AURKB-mediated phosphorylation driving release from chromosome-associated RNA. *In vitro* in interphase this results in PLD-driven CIZ1 aggregation.

To allow evaluation of the effect of the C-terminal tail on interaction with RNA, we expressed, purified, and partially characterized a series of human (C179) and mouse (C181)-derived proteins (Fig. 6A and Supplementary Fig. 4A). Size exclusion chromatography (SEC) indicated that WT forms of both species exist in a stable multimeric state (Supplementary Fig. 4). SEC coupled with multiple angle laser light scattering (SEC-MALLS) confirmed that both are homodimers with molecular weight estimates of ~44 kDa, consistent with dimerization of the ∼20 kDa monomer (Fig. 6B–D). Deletion of the C-terminal 38/37 amino acids from either the mouse or human proteins, or phosphomimetic (DDD) mutation of the human protein, had minimal effect on elution time in SEC, and SEC-MALLS confirmed human hC179_Δtail_ to still be a stable dimer (Fig. 6B). However, deletion of the MH3 domain significantly delayed SEC elution, and SEC-MALLS verified the ΔMH3 mutant is a monomer (Fig. 6C and D), indicating that the MH3 domain is required for dimerization. Modelling of secondary structure of human and murine CIZ1 MH3 domain using AlphaFold2 [47, 48] to form a dimeric entity highlighted a conserved β-strand as the putative dimerization interface (Fig. 6E). Individual full SEC and SEC-MALLS traces are given in Supplementary Fig. S4.

### Direct interaction with RNA via the C-terminal tail

Electrophoretic mobility shift assays (EMSA) have shown that murine CIZ1 (C181) can interact directly with RNAs, including *Xist* repeat E and GAPDH [9]. Here, we show that this capability is lost in the ΔMH3 mutant (Supplementary Fig. S4C), suggesting that dimerization may be involved in the creation of a stable RNA interaction interface. Furthermore, using the human WT version (C179), we confirmed direct interaction with RNA and showed this to be mediated by the tails (Fig. 6F). WT C179 shifts 99% of *Xist* probe when used at 5 µM, while the phosphomimetic mutant (C179_DDD_) shifts only 54% and the tail deletion mutant (C179_Δtail_) only 2%, under the same conditions. Similar results were returned when GAPDH RNA was used, confirming that its affinity for RNA is not specific to *Xist* sequence and may be promiscuous. Together these results confirm direct interaction with RNA, identify the unstructured C-terminal tail region of CIZ1 as an RNA-binding sequence, and show that interaction is likely modulated by phosphorylation of sites that are subject to cell-cycle-dependent regulation.

### Modelling the CIZ1 dimer

In a longer polypeptide (human C306, Fig. 6A), encompassing the three conserved zinc fingers, the MH3 domains form a tight dimer, with exposed acidic patches and unstructured C-terminal ‘tails’ that protrude from the core, with the AURKB sites available for interaction (Fig. 6G). Thus, AlphaFold 2 predictions are consistent with our experimental findings and contribute to a model in which the C-terminal tails of CIZ1 are normally embedded in chromatin via direct interaction with chromosome-associated RNAs, including *Xist*. Dissociation, driven by AURKB in mitosis, leads to CIZ1 assembly dissolution and scheduled exposure of underlying chromatin (Fig. 6H and Supplementary Fig. S4G).

## Discussion

Early concepts of a stable network of RNA-dependent proteins that form a nucleus-wide matrix have shifted to a more dynamic model in which RNAs seed localized protein assemblies [2]. Non-coding RNAs are integral to the formation of a range of membrane-less structures within the nucleus, including the nucleolus, the Xi territory and other RNA-chromatin compartments [50], and more recently, 3D maps have revealed how ncRNA-mediated recruitment of proteins to sites of transcription can regulate chromatin state [51]. However, the extent to which these RNA-localized functional assemblies are cross-linked into a wider network is not known, and may in fact vary with cell type and cell state. Most of the analysis described here is carried out in cycling primary fibroblasts, in which the CIZ1–RNA assemblies that gather around the Xi are rapidly dispersed and rebuilt each cell cycle. This study does not necessarily shed light, therefore, on their characteristics in differentiated and largely quiescent tissues.

Together with earlier analysis of the low-complexity prion-like domains (PLDs) in the N-terminal half of CIZ1 [9], the present study validates CIZ1 as a multivalent RNA-binding protein. We show that CIZ1 molecules form stable homodimers *in vitro* dependent on a highly structured MH3 domain and that while both N- and C-terminal RNA interaction interfaces are required to form large protein assemblies at the Xi [9], modulation of the one in the dual C-terminal tails is sufficient to disrupt accumulation at the Xi, as well as anchorage elsewhere in the nucleus. Moreover, under conditions in which anchorage is impaired, CIZ1 coalesces into abnormally large aggregates, likely driven by its PLDs. This suggests that anchorage creates fixed points of coalescence that restrict and limit the number of molecules that are free to aggregate at any one location.

In addition to those that gather within the Xi territory, CIZ1 forms more than a hundred smaller assemblies across the nucleus likely seeded by lncRNAs other than *Xist*. While the full scale of its RNA interactome is not known, altered expression of CIZ1 in early-stage breast cancers has been shown to disproportionately affect the levels of lncRNAs, so defining a candidate human cohort [10]. Thus, the phosphorylation-regulated dissociation of CIZ1 from RNA reported in the present study may be relevant to autosomal assemblies in addition to those at the Xi. Certainly, the consequences of CIZ1 loss or disruption are felt across the genome [8, 10], and its direct interaction with RNA is not limited to *Xist*. We postulate that AURKB phosphorylation could drive a wider dissociation of CIZ1–RNA contacts across the nucleus via a direct effect on its tails.

Phosphorylation regulates multiple steps in mitosis, from spindle formation [52] to nuclear envelope deconstruction [53–55], and its deregulation is implicated in disease states. AURKB is overexpressed in lung [56], breast [57], and prostate cancers [58] and is associated with chromosome mis-segregation leading to aneuploidy [59]. The nuclear matrix protein SAFA is also phosphorylated by AURKB during mitosis, and failure of AURKB-dependent disassembly and removal of chromatin-associated RNAs during prophase is mechanistically implicated in elevated rates of anaphase segregation defects and reduced fidelity of chromosome segregation [33]. In our studies, while we have shown that CIZ1 is required for high-fidelity chromatid division, it is not yet clear how these observations relate to the regulated behaviour of SAFA. Our data does not directly implicate failure to remove chromatin-associated RNAs, however the links between CIZ1 and cancer, both in murine models [5, 60] and *in vivo* in humans [10, 61–64], should be considered in the light of aberrant mitoses, as well as the defects in epigenetic control of gene expression that we reported previously [8].

## Supplementary Material

gkag018_Supplemental_Files

## Data Availability

Raw mass spectrometry data and proteomic results files are referenced in ProteomeXchange (PXD067610) and can be obtained from MassIVE (MSV000098911, [doi:10.25345/C51Z4259D]).

## Notes

Present address: University of Sheffield, Sheffield, S10 2TN, United Kingdom

Present address: Department of Clinical and Experimental Medicine, Brighton and Sussex Medical School, University of Sussex, Brighton BN1 9PX, United Kingdom

Present address: School of Biological Sciences, University of East Anglia, Norwich NR4 7TJ, United Kingdom

Present address: LabCorp at Otley Road, Harrogate HG3 1PY, United Kingdom

Present address: BioFrontiers Institute, University of Colorado Boulder, Boulder, CO 80303, United States
