# Supplementary material for: AURKB-driven dissolution of CIZ1–RNA assemblies from the inactive X chromosome in mitosis
Source: Nucleic Acids Res. 2026 Feb 2;54(3):gkag018. doi: 10.1093/nar/gkag018 (PMC12862363; doi:10.1093/nar/gkag018)
Supplement: gkag018_Supplemental_Files [file gkag018_supplemental_files.zip › Revised Byrom et al Supp file .pdf]

AURKB-driven dissolution of CIZ1-RNA assemblies from the inactive X chromosome in mitosis

Lewis Byrom[1,2], Gabrielle L. Turvey[1,2], Adam A. Dowle[3], Megan Thomas[1], Navin Shirodkar[1,4], Ben J. Green[1,5], Maxwell Brown[1,6], Charlotte Ball[1], Kate E. Chapman[1], Elena Guglielmi[1,2], William Dickson[1,7], Emma Noon[1], Sajad Sofi[1,8], Justin F-X. Ainscough[1,2], Alfred A. Antson[9] and Dawn Coverley[1,2]

Supplementary information

Supplementary Fig.1

A) Predicted phosphorylation sites

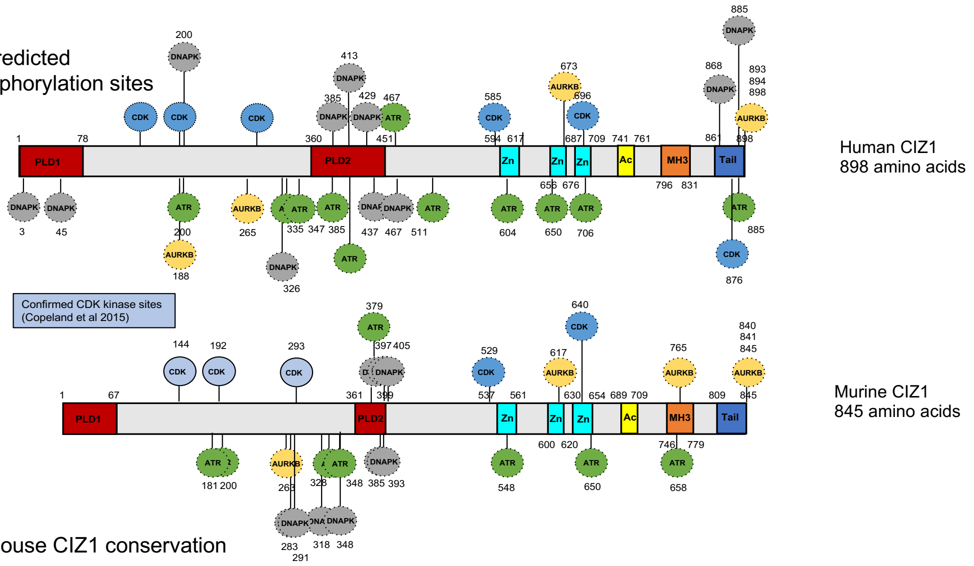

B) Human Mouse CIZ1 conservation

Domain	Identity
PLD1	77.78%
PLD2	71.43%
Zn1	91.30%
Zn2	85.71%
Zn3	95.65%
Acidic	66.67%
MH3	79.41%
Tail	60.61%
Overall	64.46%

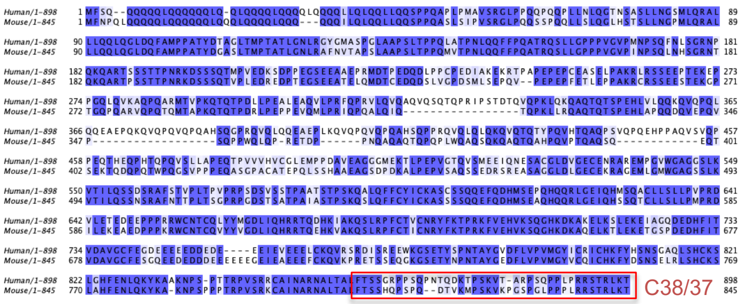

C. Detection of Xi SMACs by CIZ1 antibodies

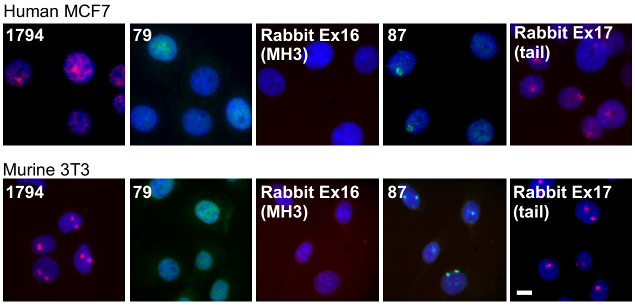

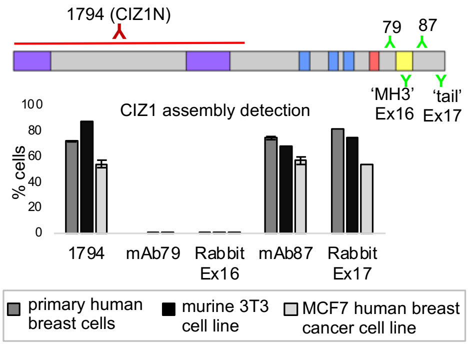

D) Disassembly of CIZ1 is accompanied by loss of Xist

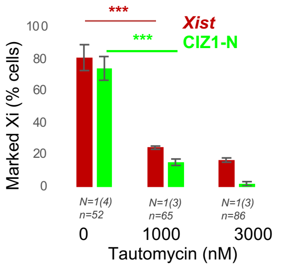

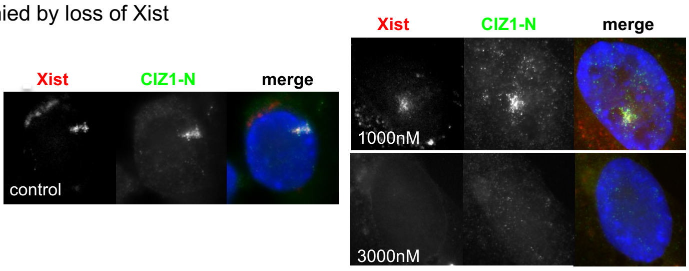

SFig.1 Conservation, functional sites, epitope availability and *Xist* release.

A) Putative phosphorylation sites displayed on full length human (Uniprot ID: Q9ULV3-1) and murine (Uniprot ID: Q8VEH2) CIZ1. Data derived from Group-based prediction System (GPS) 5.0 software (1). Consensus AURKB sites (2) are shown in yellow. Functionally relevant CDK phosphorylation sites that were experimentally determined in murine CIZ1 are shown in lighter blue (3).

B) Amino-acid alignment, right, showing high primary sequence identity between human and murine CIZ1, with domains in the table, left, created using Jalview 2.11.3.2 software with Tcoffee presets. The C-terminal 'tail' 38/37 amino acids are boxed in red, showing that the C-terminal 8 amino acids bearing putative AURKB phosphorylation sites are fully conserved.

C) Right, histogram shows frequency of CIZ1 assemblies at Xi in cycling cell populations, comparing five anti-CIZ1 antibodies by immunofluorescence. Above, summary of location of epitopes in relation to CIZ1 domains. Left, example immunofluorescence images showing epitope availability of CIZ1 in Xi assemblies in murine (3T3), and female human (MCF7) cells.

D) RNA Immuno-FISH for CIZ1 N and *Xist,* with and without Tautomycin for 15 hours. Histogram shows the proportion of cells in a cycling population that retain CIZ1 or *Xist* at Xi (including tight and dispersed clouds). Right, representative images showing *Xist* (red) and CIZ1-N (green) in D3T3 cells. For 1uM Tautomycin an example of a partially dispersed *Xist* cloud is shown.

Supplementary Fig.2

A. Differential detection of CIZ1 epitopes in metaphase

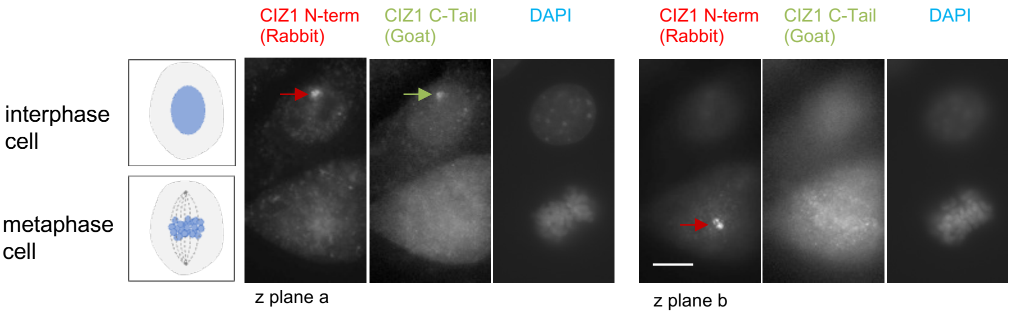

B. Summary of in vitro modified sites

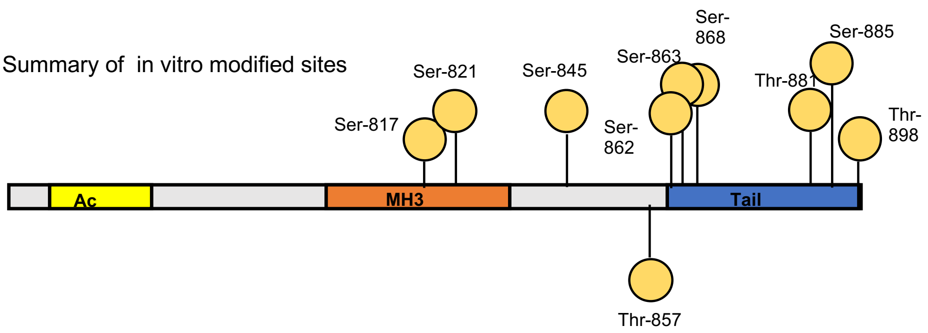

C. High confidence phosphopeptides identified by TimsTOF

Phosphorylated residue	Phospho-peptides	Site localisation probablility	Peptide Intensity (-) Kinase	Peptide Intensity (+) Kinase	Percentage intensity (+) Kinase / (-) Kinase
Y782	GSETySPNTAYGVDFLVPVMGYICR	33%	0.00E+00	4.34E+04	100%
S810	FYHsNSGAQLSHCK	40%	0.00E+00	1.30E+04	100%
S812	FYHSNsGAQLSHCK	88%	8.91E+04	2.21E+05	71%
S817	FYHSNSGAQLsHCK;FYHSNSGAQLsHCKsLGHFENLQK	96%	0.00E+00	1.54E+05	100%
S821	FYHSNSGAQLSHCKsLGHFENLQK;sLGHFENLQK	100%	4.78E+04	3.43E+06	99%
S845	NPSPTTRPVsR;NPSPTTRPVsRR	97%	0.00E+00	2.31E+06	100%
T857	NALtALFTSSGRPPSQPNTQDK;NALtALFTSSGRPPSQPNTQDKTPSK;NALtALFTSSGRPPSQPNtQDKTPSK;NALtALFTSSGRPPsQPNTQDK	97%	2.73E+04	7.13E+06	100%
T861	NALTALFtSSGRPPSQPNTQDK	62%	3.83E+04	1.45E+06	97%
S862	NALTALFTsSGRPPSQPNTQDK;NALTALFTsSGRPPSQPNTQDKTPSK	97%	4.30E+05	8.84E+06	95%
S863	NALTALFTSsGRPPSQPNTQDK;NALTALFTSsGRPPSQPNTQDKTPSK;NALTALFTSsGRPPSQPNtQDK;NALTALFTSsGRPPsQPNTQDKTPSK	97%	4.84E+05	1.11E+07	96%
S868	NALTALFTSSGRPPsQPNTQDK;NALTALFTSSGRPPsQPNTQDKTPSK;NALTALFTSsGRPPsQPNTQDKTPSK;NALtALFTSSGRPPsQPNTQDK	97%	1.82E+05	3.24E+07	99%
T872	NALTALFTSsGRPPSQPNtQDK;NALtALFTSSGRPPSQPNtQDKTPSK	73%	0.00E+00	2.97E+05	100%
T876	NALTALFTSSGRPPSQPNTQDKtPSK;NALTALFTSSGRPPSQPNtQDKtPSK;NALTALFtSSGRPPSQPNTQDKtPSK	93%	3.50E+05	7.56E+04	18%
S878	NALTALFTSSGRPPSQPNTQDKTPsK	77%	1.16E+05	4.21E+04	27%
T881	VtARPSQPPLPR;VtARPsQPPLPR	100%	0.00E+00	1.46E+06	100%
S885	PsQPPLPR;VTARPsQPPLPR;VtARPsQPPLPR	100%	9.72E+04	1.06E+08	100%
T898	KTPSKVTARPsQPPLPRRSTRLKt	84%	0.00E+00	1.14E+05	100%

SFig.2 AURKB site epitope availability, and in vitro modified epitopes

A) Example images of neighbouring interphase and metaphase D3T3 cells co-stained for CIZ1-C using a different anti-peptide antibody raised against the tail region encompassing the AURKB kinase site cluster (E17 raised in goat), and CIZ1-N (1794). Two focal planes are shown, revealing both epitopes in the interphase cell (left) but only the CIZ1-N epitope in the metaphase cell. DNA is stained with Dapi. Bar is 10 microns.

B) Summary of *in vitro* modified phosphorylation sites showing ten determined sites detected in hCIZ1C179 treated with purified AURKB.

C) Table shows identified sites reported from phosphopeptides with ≥95% relative abundance compared to untreated control, and ≥75% site-localisation probability. The phosphopeptides for positive sites/residues are reported in format of lower-case, where t or s is phosphorylated. Residues in reference to full-length human CIZ1 Q9ULV3.

Supplementary Fig.3

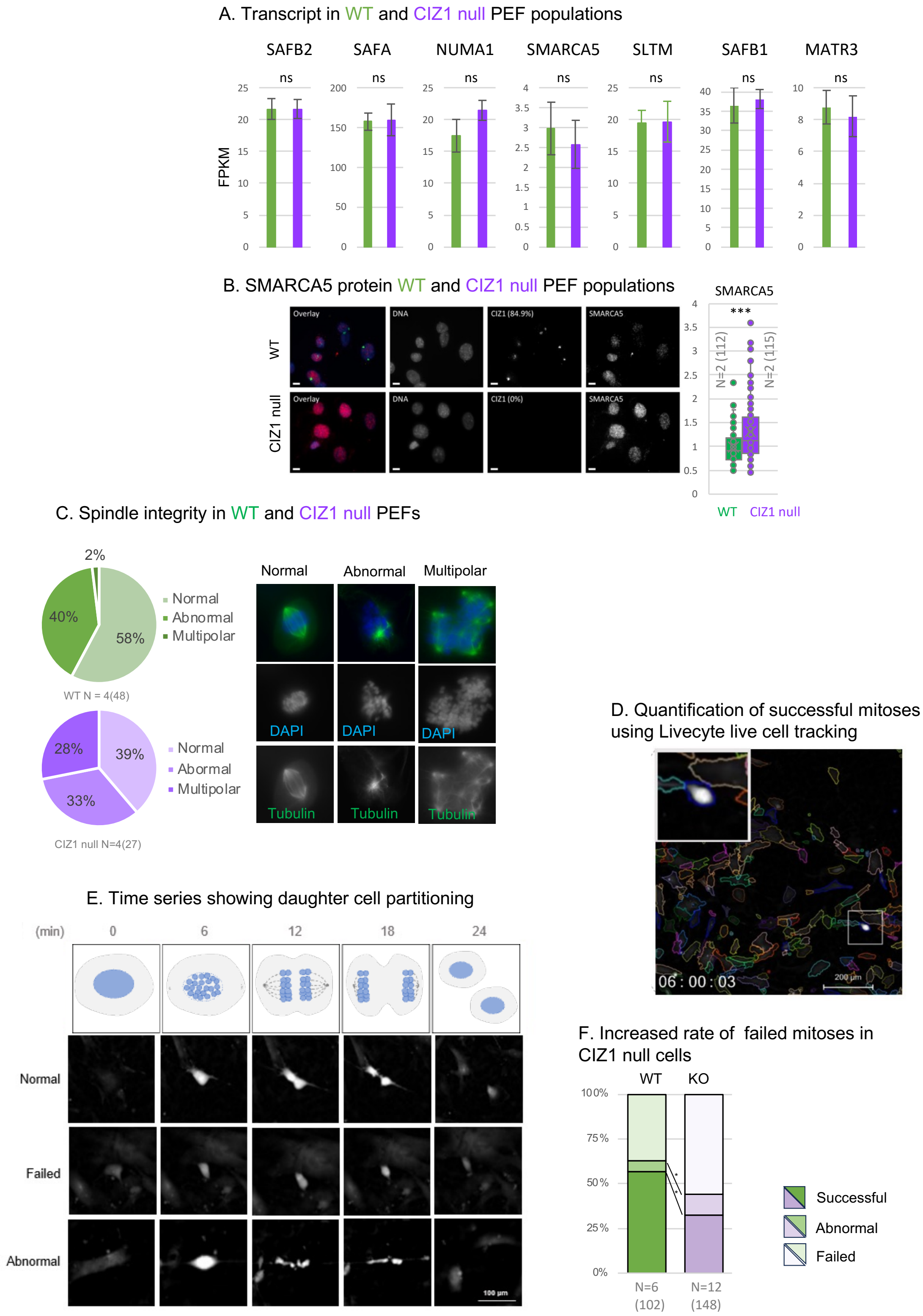

SFig.3 Nuclear matrix proteins and mitotic defects in CIZ1 KO cells.

A) Relative transcript levels in triplicate populations of WT and CIZ1 null PEFs (4), showing no significant difference in expression.

B) Example immunofluorescence images of SMARCA5 (red) in cycling WT and CIZ1 null PEFs, co-stained for CIZ1-C (green), and Dapi (blue). Box and whisker plot shows intensity measures derived from two independent primary cell populations (N) for each genotype. N=number of nuclei measured. Comparison is by t-test where *** denotes p<0.001, and indicates a significant elevation of SMARCA5 epitope in CIZ1 null cells.

C) Classification of mitotic cells into three groups: cells with visually normal bipolar mitotic spindles, cellsl with abnormal bipolar spindles or aberrant position, or cells with multipolar spindles. Data is expressed as % for mitotic cells derived from four WT and four CIZ1 null asynchronous PEF populations. Below, images show examples of cells stained for alpha-tubulin (ab7291, green), with normal and abnormal spindles.

D) Example frame from Livecyte image analysis, monitoring morphological differences and mitotic events in populations of WT and CIZ1 null PEFs, with inset showing a cell undergoing mitosis. Three viewpoints were selected per well for image acquisition over 24 hours.

E) Example images showing mitotic events that were either normal (giving rise to two daughter cells), abnormal (uneven products), or failed (no separation), seen via timelapse.

F) Histogram showing frequency of each class. CIZ1 null cells have significantly increased rate of failed mitoses, where * denotes p<0.05 (t-test).

Supplementary Fig.4

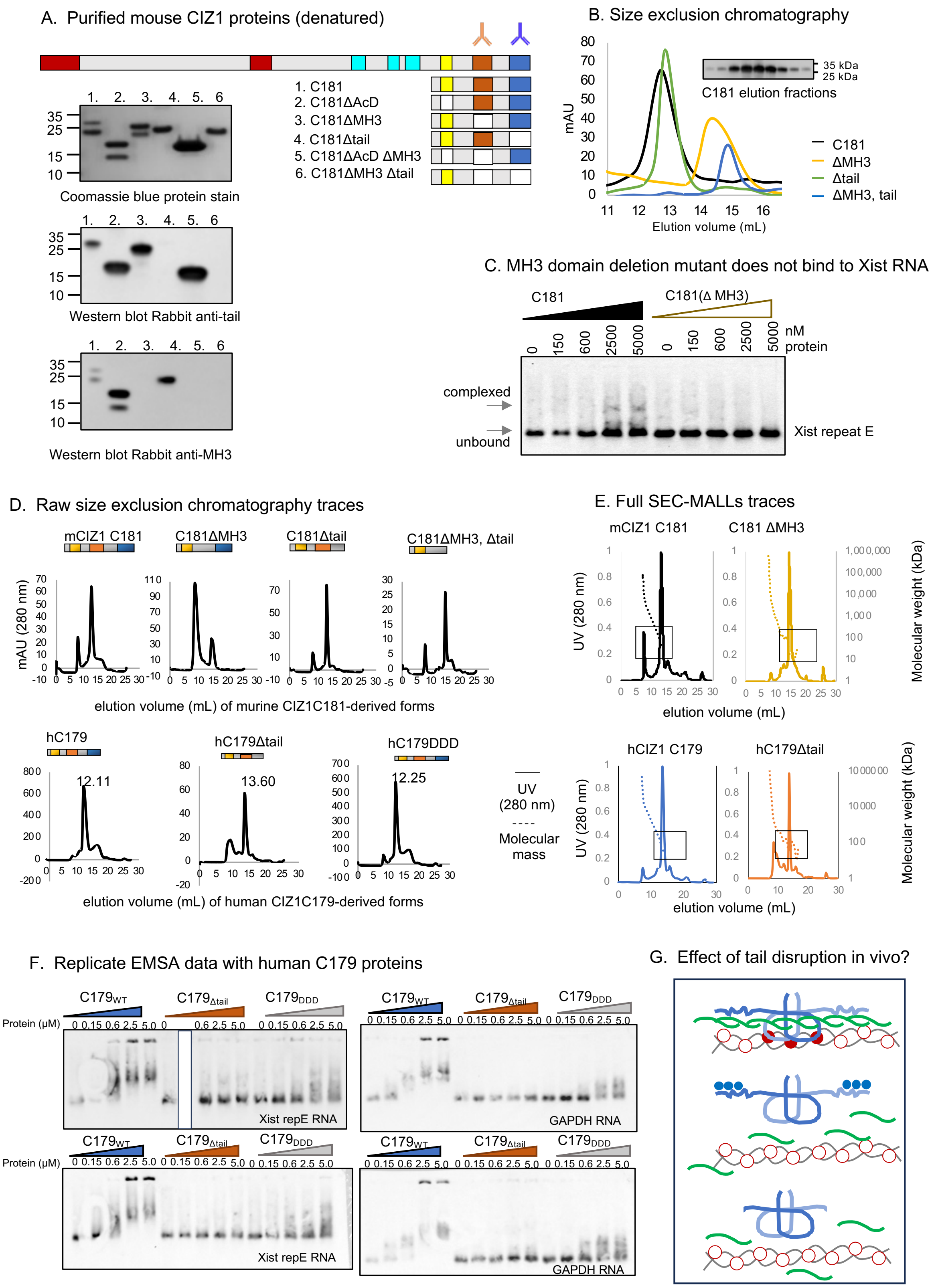

SFig.4 Murine CIZ1 C181-derived proteins, additional EMSA and SEC analysis

A) Murine C181-derived expression constructs. Left, expressed and purified proteins shown after denaturation in SDS-PAGE. Upper gel is stained with coomassie blue to reveal all protein isoforms, and shows the tendency of constructs bearing the 38 amino acid tail domain to produce two forms. Centre, anti-tail antibody fails to detect the smaller of the two in western blot, suggesting that the tail domain is cleaved during expression. Lower, anti-MH3 domain antibody fails to detect MH3 domain deletion mutants, but does detect cleaved and uncleaved forms in which MH3 domain is present.

B) SEC profiles for the indicated murine proteins. Inset shows western blot of elution fractions for C181, probed with anti-tail antibody.

C) EMSA showing interaction between murine CIZ1-C181$_{WT}$ protein and *Xist* repeat E RNA probe, compared to that with murine CIZ1-C181$_{\Delta MH3}$.

D) Upper, full SEC chromatograms for purifiied mCIZ1-C181$_{WT}$, mCIZ1-C181$_{\Delta MH3}$, mCIZ1-C181$_{\Delta tail}$ and mCIZ1-C181$_{\Delta MH3 \Delta tail}$. Lower, Full SEC chromatograms for purification of hCIZ1-C179$_{WT}$, hCIZ1-C179$_{\Delta tail}$, and hCIZ1-C179$_{DDD}$.

E) Upper, full SEC-MALLS chromatograms showing normalised UV absorbance at 280nm, and molar mass (dotted line) for mCIZ1-C181$_{WT, and}$ mCIZ1-C181$_{\Delta MH3.}$ Lower, full SEC-MALLS chromatograms for of hCIZ1-C179$_{WT}$, and hCIZ1-C179$_{\Delta tail}$. Boxes indicating where focused versions of graphs are displayed in Fig 6.

F) Replicate EMSAs showing *Xist* repeat E (left) and GAPDH (right) RNA interaction with hCIZ1-C179$_{WT}$, and impaired interaction with hCIZ1-C179$_{\Delta tail}$ or hCIZ1-C179$_{DDD}$. Data, incorporated into quantification shown in Fig.6F.

G) Extended model from Fig.6H, depicting CIZ1 homodimers interacting with chromosome-associated RNAs via its C-terminal tails (upper), and the effect of phosphomimetic tail mutation (middle) or tail deletion (lower).

Supplemental dataset 1 (Xl). Related to Figures 4 and 5.
Results of three protein interaction studies.
Tab 1 Raw results study A
Tab 2 Raw results study B
Tab 3 Raw results study C

Supplemental dataset 2. Related to Figures 4 and 5.
Analysis of three protein interaction studies.
Tab 1 Core 56 interacting proteins
Tab 2 GSEA cluster analysis in STRING
Tab 3 *Xist* interaction overlap
Tab 4 WT compared to tail deletion mutant
Tab 5 WT compared to phosphomimetic mutant

References to supplementary information

1. Wang, C., Xu, H., Lin, S., Deng, W., Zhou, J., Zhang, Y., Shi, Y., Peng, D. and Xue, Y. (2020) GPS 5.0: An Update on the Prediction of Kinase-specific Phosphorylation Sites in Proteins. *Genomics Proteomics Bioinformatics*, **18**, 72-80.
2. Kettenbach, A.N., Schweppe, D.K., Faherty, B.K., Pechenick, D., Pletnev, A.A. and Gerber, S.A. (2011) Quantitative phosphoproteomics identifies substrates and functional modules of Aurora and Polo-like kinase activities in mitotic cells. *Sci Signal*, **4**, rs5.
3. Copeland, N.A., Sercombe, H.E., Wilson, R.H. and Coverley, D. (2015) Cyclin-A-CDK2-mediated phosphorylation of CIZ1 blocks replisome formation and initiation of mammalian DNA replication. *J Cell Sci*, **128**, 1518-1527.
4. Stewart, E.R., Turner, R.M.L., Newling, K., Ridings-Figueroa, R., Scott, V., Ashton, P.D., Ainscough, J.F.X. and Coverley, D. (2019) Maintenance of epigenetic landscape requires CIZ1 and is corrupted in differentiated fibroblasts in long-term culture. *Nat Commun*, **10**, 460.
